# EGFR inhibition augments the therapeutic efficacy of the NAT10 inhibitor Remodelin in Colorectal cancer

**DOI:** 10.1186/s13046-025-03277-y

**Published:** 2025-02-04

**Authors:** Yongbin Zheng, Dan Song, Ming Guo, Chenhong Wang, Mingzhen Ma, Gongcai Tao, Licui Liu, Xiaobo He, Fengyu Cao, Dan Luo, Qingchuan Zhao, Zhongyuan Xia, Yanxin An

**Affiliations:** 1 Department of Gastrointestinal Surgery, Key Laboratory of Hubei Province for Digestive System Disease, Renmin Hospital of Wuhan University, Wuhan, Hubei 430000 China; 2 Department of Anesthesiology, Renmin Hospital of Wuhan University, Wuhan, Hubei 430000 China; 3 Department of Gastroenterology, Minda Hospital of Hubei Minzu University, Enshi, 445000 China; 4 State Key Laboratory of Cancer Biology, National Clinical Research Center for Digestive Diseases and Xijing Hospital of Digestive Diseases, Fourth Military Medical University, Xi’an, Shaanxi Province 710032 China; 5 Department of General Surgery, The First Affiliated Hospital of Xi ’an Medical University, No. 48 Fenghao West Road, Lianhu District, Xi ’an, Shaanxi Province 710077 China

**Keywords:** N-acetyltransferase 10, Colorectal cancer, Cetuximab, Remodelin

## Abstract

**Background:**

Colorectal cancer (CRC) is the second leading cause of cancer-related death worldwide, and treatment options for advanced CRC are limited. The regulatory mechanisms of aberrant NAT10-mediated N4-acetylcytidine (ac4C) modifications in cancer progression remains poorly understood. Consequently, an integrated transcriptomic analysis is necessary to fully elucidate the role of NAT10-mediated ac4C modifications in CRC progression.

**Methods:**

NAT10 expression levels were analyzed in CRC samples and compared with those in corresponding normal tissues. The potential mechanisms of NAT10 in CRC were investigated using RNA sequencing, RNA immunoprecipitation sequencing, and acetylated RNA immunoprecipitation sequencing. Additional in vivo and in vitro experiments, including CCK-8 assays, colony formation and mouse xenograft models, were conducted to explore the biological role of NAT10-mediated ac4C modifications. We also evaluated and optimized a potential treatment strategy targeting NAT10.

**Results:**

We found that NAT10 is highly expressed in CRC samples and plays a pro-oncogenic role. NAT10 knockdown led to PI3K-AKT pathway inactivation, thereby inhibiting CRC progression. However, treatment with the NAT10 inhibitor Remodelin induced only a limited and reversible growth arrest in CRC cells. Further epigenetic and transcriptomic analysis revealed that NAT10 enhances the stability of ERRFI1 mRNA by binding to its coding sequence region in an ac4C-dependent manner. NAT10 knockdown decreased ERRFI1 expression, which subsequently activated the EGFR pathway and counteracted the inhibitory effects on CRC. Based on these findings, we demonstrated that dual inhibition of NAT10 and EGFR using Remodelin and the EGFR-specific monoclonal antibody cetuximab resulted in improved therapeutic efficacy compared to either drug alone. Moreover, we observed that 5-Fluorouracil promoted the interaction between NAT10 and UBR5, which increased the ubiquitin-mediated degradation of NAT10, leading to ERRFI1 downregulation and EGFR reactivation. Triple therapy with Remodelin, cetuximab, and 5-Fluorouracil enhanced tumor regression in xenograft mouse models of CRC with wild-type KRAS, NRAS and BRAF.

**Conclusions:**

Our study elucidated the mechanism underlying 5-Fu-induced NAT10 downregulation, revealing that NAT10 inhibition destabilizes ERRFI1 mRNA through ac4C modifications, subsequently resulting in EGFR reactivation. A triple therapy regimen of Remodelin, cetuximab, and 5-Fu showed potential as a treatment strategy for CRC with wild-type KRAS, NRAS and BRAF.

**Supplementary Information:**

The online version contains supplementary material available at 10.1186/s13046-025-03277-y.

## Background

Colorectal cancer (CRC) is the second leading cause of cancer-related death worldwide [1]. While most patients with early-stage CRC can be cured with surgery, the survival rate for patients with metastatic CRC remains low [2]. Standardized chemotherapy and targeted therapies have improved outcomes. However, few patients experience long-term benefits due to drug resistance that arises before or during treatment [3]. Consequently, developing effective strategies to enhance chemo-sensitivity and identifying novel molecular therapy targets are major research priorities.

N-acetyltransferase 10 (NAT10), the only known ac4C “writer” protein, is a key regulator of RNA metabolism and implicated in cancer progression [4, 5]. Recent studies have underscored the therapeutic potential of the NAT10 inhibitor Remodelin, which can reverse drug resistance and increase apoptosis across various cancer cell types [5]. For example, Remodelin has been shown to inactivate the Wnt/β-catenin signaling pathway by destabilizing *KIF23* mRNA, thereby inhibiting CRC cell proliferation [6]. However, due to the substantial genetic variability in CRC, the specific patient subsets that might benefit from Remodelin treatment have not yet been established. Therefore, it is critical to integrate transcriptomic, genetic, and epigenetic data to better understand NAT10 and maximize the therapeutic efficacy of Remodelin in CRC. Additionally, NAT10-mediated ac4C modification facilitates adaptive stress responses in cancer cells to drugs and other extracellular stimuli. For instance, NAT10 is induced by Helicobacter pylori infection and stabilizes *MDM2* mRNA via RNA acetylation to promote gastric cancer progression [7]. Mapping ac4C-modified RNA substrates under stress conditions, especially during chemotherapy, may identify targets for optimizing the therapeutic effects of Remodelin in CRC.

The partial activation of redundant downstream signaling pathways is a primary cause of tumor relapse following an initial response to targeted therapy. For example, ERRFI1 downregulation by MRTX1133 (a KRAS^G12D^ inhibitor) leads to EGFR activation and subsequent MRTX1133 resistance in KRAS^G12D^-mutant CRC cells [8]. Therefore, elucidating the mechanisms underlying ERRFI1 dysregulation can help optimize treatment strategies. Previous studies have demonstrated that ERRFI1 can be induced by a variety of extracellular and intracellular stimuli, as its promoter region contains multiple regulatory elements [9]. Furthermore, ERRFI1 is regulated by several miRNAs at the post-transcriptional level and is subject to post-translational phosphorylation [10, 11]. However, it remains unclear whether RNA modification sites exist on *ERRFI1* transcripts, and if so, how such modifications might affect expression or the efficacy of targeted therapies.

In this study, we evaluated the effects and mechanisms of action of NAT10, as well as the therapeutic efficacy of Remodelin in CRC. Although NAT10 knockdown inactivated the PI3K-AKT pathway and inhibited CRC progression to some extent, Remodelin treatment alone induced only reversible growth arrest in CRC cells and demonstrated limited therapeutic efficacy. To investigate the mechanisms underlying NAT10’s effects, we performed transcriptome-wide acetylated RNA immunoprecipitation sequencing (acRIPseq), RNA immunoprecipitation sequencing (RIP-Seq), and molecular experiments. Our results indicated that ERRFI1 is a direct target of NAT10, and NAT10 silencing reduces the stability of ERRFI1 mRNA by decreasing binding to the coding sequence (CDS) region of ERRFI1 mRNA. This reduction in stability subsequently activated the EGFR pathway, counteracting the inhibitory effects on CRC. Accordingly, we evaluated the effects of combining Remodelin with the EGFR-specific monoclonal antibody cetuximab, both in vitro and in vivo, as well as the impact of 5-Fluorouracil (5-Fu) treatment on NAT10, UBR5 and ERRFI1. Collectively, our results demonstrate that 5-Fu-induced NAT10 downregulation and the subsequent regulation of ERRFI1 ac4C modification provide insights into NAT10’s role in CRC progression, potentially contributing to the development of improved CRC treatment strategies.

## Methods

### Cell culture and reagents

Human CRC cell lines, including CACO2, SW48, DLD1, SW480, SW620, and HCT-116, were purchased from Procell Life Science & Technology Company (Wuhan, China). SW48 cells were maintained in high-glucose Dulbecco’s modified Eagle’s medium (DMEM; Procell, PM150210B), and HCT-116 cells were cultured in McCoy’s 5 A medium supplemented with 50 U/mL penicillin-streptomycin (Biosharp, Hefei, China) and 10% FBS (Vivacell, Shanghai, China). CACO2 cells were cultured in Minimum Essential Medium (MEM; Procell, China) with 20% FBS and 1% non-essential amino acid (NEAA; Gibco, Waltham, MA, USA), while DLD1 cells were incubated in RPMI-1640 medium (Biosharp, Hefei, China) containing 10% FBS. SW480 and SW620 cells were grown in Leibovitz’s L-15 medium (Biosharp, Hefei, China) supplemented with 10% FBS. All cells were grown as monolayers on plastic culture dishes at 37 °C in 5% CO2 in a humidified incubator. Once cells reached 70–80% confluence, they were treated with 0.05% trypsin (Gibco, Waltham, MA, USA), centrifuged at room temperature for 3 min at 1100 rpm, and resuspended in fresh culture media in a new flask. Reagents used in this study are listed in Supplementary Table 1.

### Tumor samples

Fresh CRC and surrounding tissue specimens were obtained from patients who had undergone surgery for CRC at Xijing Hospital of Digestive Diseases. The specimens were snap-frozen in liquid nitrogen immediately after collection. All specimens were pathologically and clinically labeled. Hematoxylin-eosin staining was conducted at the Department of Pathology of Xijing Hospital to confirm the histomorphology of all primary tumor samples and regional lymph nodes. The Xijing Hospital’s Protection of Human Subjects Committee approved the study, and all participants provided informed consent.

### Construction of lentiviral cell lines and plasmids transfection

To construct stable cancer cell lines with targeted protein knockdown or overexpression, lentiviruses carrying shRNA for the indicated protein or the targeted protein-coding sequences were constructed by GENECHEM (Shanghai, China). The shRNA sequences were as follows: shNAT10-1: cgCAAAGTTGTGAAGCTATTT; shNAT10-2: cgAGCTGGATTTGTTCCTGTT; shERRFI1-1: GGATATCCAACTGTTGTAT; shERRFI1-2: TATGGTTGCCAAGTAACCTGC; shUBR5-1: CAACTTAGATCTCCTGAAA; shUBR5-2: GCTGGCAGGCAACACCTTAGG. The cell lines were infected with lentiviruses in 6-well plates according to the manufacturer’s instructions. Sixteen hours after transfection, the medium was replaced with complete medium. Once the cells reached 70–80% confluence, they were transferred to T25 flasks and selected by incubation with 2 µg/mL puromycin for 2 weeks. The knockdown or overexpression efficiency was verified using RT-qPCR and western blotting (WB). CRC cells were also transfected with HA/FLAG/MYC-tagged wild-type or mutated full-length plasmids according to the manufacturer’s instructions using Lipofectamine 8000 transfection reagent (Beyotime). WB and RT-qPCR were performed 48 h post-transfection.

### Western blotting analysis

WB was performed as described previously [12]. Primary antibodies against GAPDH, ACTIN, TUBULIN, NAT10, ERRFI1, EGFR, p-EGFR, ERK, p-ERK, PI3K, p-PI3K, AKT, p-AKT, mTOR, p-mTOR, STAT1, p-STAT1, UBR5, UB, FLAG, HA, and MYC were used. A complete list of these antibodies is shown in Supplementary Table 2. Immunoreactivity was visualized using a Chemiluminescence Detection Kit (Biosharp), and ECL signals were captured using the Bio-Rad ChemiDoc Imaging System (Hercules, CA, USA).

### Real-time quantitative PCR (RT-qPCR) analysis

Total RNA was extracted from cell lysates using TriQuick Reagent (R1100; Solarbio, Beijing, China) according to the manufacturer’s instructions. Cells were harvested and collected in 1 mL of TriQuick Reagent after being washed twice with 1 mL of PBS. Next, 200 µL of chloroform was added and the sample was vortexed for 30 s, followed by a 3-minute incubation on ice. The sample was then centrifuged for 10 min at 4℃ and 12,000 × g. The upper aqueous phase (~ 400 µL) was mixed with 500µL of isopropanol in a fresh tube, inverted 10 times, and incubated for 10 min at room temperature. The mixture was then centrifuged for 10 min at 12,000 × g, and the supernatant was carefully removed without disrupting the pellet. The RNA pellet was washed with 1 mL of 75% ethanol and centrifuged at 12,000 × g for 2 min at 4 °C. After removing the supernatant, the pellet was air-dried for 10 min. Finally, the RNA was dissolved in 50–100 µL of nuclease-free water, and the RNA concentration was measured using a NanoDrop Spectrophotometer (ND-1000; Thermo Scientific, Waltham, MA, USA).

Total RNA was reverse-transcribed using the PrimeScript RT Enzyme MIX Kit (RR037A; Takara, Kusatsu, Japan) according to the manufacturer’s instructions. RT-qPCR was performed using TB Green Premix Ex Taq II (RR820A; Takara) or SYBR Green Pro Taq HS kit (AG11701; Accurate Biology, China) and a CFX96 Touch Real-Time PCR Detection System (Bio-Rad Laboratories) or Lightcycler 4800II. Human endogenous ACTIN was used as an internal control to normalize relative mRNA levels. RT-qPCR experiments were performed in triplicate, and results were analyzed using the comparative Ct method. All primers are listed in Supplementary Table 3.

### Cell viability assays

An appropriate number of cells were seeded into 96-well plates and incubated overnight. Cells in each well, with or without the indicated drugs, were incubated with 10µL of CCK-8 reagent (Biosharp) for 2 h. Cell viability was assessed by measuring absorbance at OD450nm using a multimode plate reader (Bio-Tek Instruments, Winooski, VT, USA).

### Immunohistochemistry

Immunohistochemical(IHC) staining was performed on 5-µm-thick formalin-fixed, paraffin-embedded colon sections. Briefly, sections were deparaffinized, followed by antigen retrieval using a citrate antigen retrieval solution (G1201-1 L; Servicebio, Wuhan, China). Endogenous peroxidase blocking, secondary antibody incubation, and DAB substrate-chromogen reactions were performed using a Non-Biotinyl Detection System (mouse/rabbit) IHC Kit (PV-9000 and ZLI-9019; ZSGB-BIO, Beijing, China) according to the manufacturer’s instructions. Sections were stained with a specific primary antibody (Ki-67, 1:200; Servicebio). Counter-staining was performed using hematoxylin (ST2067; Beyotime, Shanghai, China) and an acid-alcohol fast differentiation solution (C0163; Beyotime). Finally, the stained sections were visualized under an Olympus microscope.

### RNA sequencing (RNA-seq) and data analysis

Transcriptome profiling of NAT10 KD and control SW620 cells was performed via RNA-seq at CloudSeq Biotech Co., LTD (Shanghai, China). Differential gene detection was conducted using DESeq2. A p-value < 0.05 and a fold change (FC) > 2 or < -2 were used as thresholds to identify differentially expressed genes (DEGs).

### RNA immunoprecipitation and sequencing (RIP-seq) and RIP‑qPCR

RIP-seq was conducted at CloudSeq Biotech Co., LTD. RIP-qPCR was performed according to the manufacturer’s instructions, using an RNA Immunoprecipitation Kit (P0101; GeneSeed, Guangzhou, China). Briefly, 2 × 10^7^ cells in a 10-cm dish at 75% confluency were harvested and lysed in complete RIP Lysis Buffer, supplemented with RNase A and protease inhibitor. Cell lysates were then incubated with anti-NAT10 antibody (194297; Abcam) or normal Rabbit IgG (30000-0-AP; Proteintech, Wuhan, China)-coated protein A/G beads at 4 °C overnight, using a rotation mixer at the lowest speed. RIP Wash Buffer was used to wash the magnetic bead complexes. Beads were separated from the samples using a magnetic separator, and RNA from the Input and IP groups was extracted using the phenol/chloroform method with RC Columns. Real-time RT-qPCR was performed to quantify ERRFI1 mRNA levels.

### Acetylated RNA immunoprecipitation and sequencing (acRIP-seq) and acRIP-qPCR

The acRIP-seq was conducted at CloudSeq Biotech Co., LTD. Anti-ac4C immunoprecipitation (ac4C-RIP) was performed using the GenSeq^®^ ac4C IP Kit (GS-ET-005; GenSeq, Nanjing, China), following the manufacturer’s instructions. Total RNA (120 µg) was fragmented to produce a distribution of RNA fragment sizes centered at approximately 200nt. Magnetic beads coated with either an anti-ac4C antibody or normal rabbit IgG were prepared for immunoprecipitation with the fragmented RNA mixture. After washing the beads, the eluted RNA was purified for subsequent RT-qPCR. In this assay, IgG served as a negative control.

### Identification of NAT10 ubiquitination site by mass spectrometry

The experiment was performed at PTM Biolab (Hangzhou, China). SW620 cells were rinsed with ice-cold PBS and collected in a centrifuge tube. The process was as follows: the sample was sonicated on ice after the addition of four volumes of lysis buffer (8 M urea, 1% Protease Inhibitor Cocktail). The supernatant was collected after centrifugation at 12,000 g at 4 ℃ for 10 min. Protein concentration was determined using the BCA kit according to the protocol. For digestion, proteins were precipitated by adding the sample to TCA, vortexed, incubated for 2 h at 4 ℃ and collected by centrifugation at 4500 g for 5 min at 4 ℃. The precipitated protein was washed 3 times with pre-cooled acetone and dried for 1 min before being redissolved in TEAB to a final concentration of 200 mM. Trypsin was added for digestion overnight. Dithiothreitol (DTT) was added to a final concentration of 5 mM and incubated at 56 °C for 30 min for reduction. Iodoacetamide (IAA) was then added to a final concentration of 11 mM and incubated at room temperature for 15 min in the dark. Finally, the sample was resolved in IP buffer NETN (100 mM NaCl, 1 mM EDTA, 50 mM Tris-HCl, 0.5% NP-40, pH 8.0). The supernatant was incubated with pre-washed ubiquitin antibody beads (PTM-1104, PTM Bio) at 4℃overnight, rotating at the slowest speed. The beads were washed four times with wash buffer and twice with deionized water. Finally, bound peptides were eluted three times with 0.1% trifluoroacetic acid. The eluate was collected and vacuum frozen. After extraction, salts were removed according to C18 ZipTips manufacturer instructions for liquid mass analysis. For LC-MS/MS analysis, peptides were separated by liquid chromatography in mobile phase A and separated using the Vanquish Neo ultra-efficient liquid phase system. Mobile phase A is an aqueous solution containing 0.1% formic acid; mobile phase B is an aqueous solution containing 0.1% formic acid and 80% acetonitrile. The peptides were separated by an ultra-efficient liquid phase system and injected into the NSI ion source for ionization before entering the Orbitrap Astral mass spectrometer for analysis. The data acquisition mode used the data-independent acquisition (DIA) procedure. Acquired MS/MS data were processed using the DIA-NN search engine (v.1.8). FDR was adjusted to < 1%.

### Ubiquitination assay and immunoprecipitation

Exogenous FLAG-UBR5 and MYC-UB were overexpressed in SW620 cells transfected with either wild-type or mutated HA-NAT10 plasmid for 24 h. The cells were then cultured in fresh medium containing MG132 for 8 h. The ubiquitination level of exogenous MYC-UB was assessed using a non-denaturing immunoprecipitation (IP) protocol. For IP and Co-immunoprecipitation (Co-IP), the Abcam Immunoprecipitation Kit (ab206996; Cambridge, UK) was used. Briefly, cells were washed three times with ice-cold PBS, lysed in cold lysis buffer containing a phosphatase and protease inhibitor cocktail, and incubated on ice for 1 min. The disrupted cell suspension was transferred to a chilled microcentrifuge tube, wrapped with parafilm, and placed on a rotary mixer for 30 min at 4 °C at the slowest speed. After centrifugation at 10,000 × g for 10 min, the supernatant was collected and incubated with primary antibodies. The immunoprecipitation system was supplemented with lysis buffer up to 500µL and mixed overnight at 4 °C. Next, 40µL of prepared Protein A/G Sepharose^®^ bead slurry was added for bead capture. The immunocomplexes were washed with 1× Wash Buffer, eluted in 2× SDS loading buffer by boiling for 5 min, and frozen at -80 °C for western blot analysis. For the ubiquitination assay, samples were subjected to immunoblotting with anti-MYC antibodies to visualize polyubiquitinated protein bands.

### RNA decay assay

An RNA decay assay was performed to investigate the effect of NAT10-mediated ac4C modification on ERRFI1 mRNA stability. SW620-shNC, SW620-shNAT10, DLD1-Vector, DLD1-LV-NAT10-WT, DLD1-LV-NAT10G641E, and DLD1-LV-NAT10K290A cells were cultured in 6-well plates. Actinomycin D (HY-17559; MedChemExpress, Monmouth Junction, NJ, USA) was applied at a final concentration of 5 µg/mL. Cells were collected at defined time intervals, and total RNA was isolated for RT-qPCR to quantify the relative abundance and half-life of ERRFI1 mRNA.

### Protein half-life assay

Cells were seeded into 6-well plates and treated with cycloheximide (CHX) at a final concentration of 30 µg/mL to inhibit protein synthesis. Cells were harvested at 0, 6, 12, 18, 24, or 30 h. After each time point, whole-cell lysates were prepared, and the expression levels of NAT10 were quantified.

### Colony formation assay

Cells were seeded into 6-well plates at appropriate densities and incubated for 10–15 days. The colonies formed were fixed with 4% paraformaldehyde for 30 min and then stained with 1% methylthionine chloride for 10 min, and analyzed using ImageJ software.

### Cell cycle assay

The distribution of cell cycle phases was assessed using a cell cycle staining kit (CCS012; Multi Sciences, Hangzhou, China) and flow cytometry. Colon cancer cells from 6-well plates were collected, washed, and resuspended in 1 mL of DNA staining solution with 10 µL of permeabilization solution. The samples were gently vortexed and incubated at room temperature in the dark for 30 min. The cell cycle distribution was then analyzed using a FACS Canto II instrument (BD Biosciences, Franklin Lakes, NJ, USA) and FlowJo 10 software.

### EdU assay

Cell proliferation was evaluated using the Cell Light EdU DNA Imaging Kit (R11053.9; RiboBio, Guangzhou, China) following the manufacturer’s instructions. EdU incorporation was measured by adding EdU to the culture media at a final concentration of 10 μM at 37℃ for 2 h. Fluorescent images were captured with an Olympus fluorescence microscope (Olympus, Tokyo, Japan) and cells were counted from three randomly selected fields using ImageJ.

### Flow cytometric assay

To detect apoptotic cell death, cells from 6-well plates were washed with PBS and stained using the Annexin V-APC7-AAD apoptosis kit (AT105-01; MultiSciences), following the manufacturer’s protocol. After 15 min of incubation in the dark at room temperature, samples were analyzed using flow cytometry. Data analysis was performed using FlowJo 10 software to determine the percentage of positive cells for each tube.

### Acetylation site prediction

The PACES (http://rnanut.net/paces/) was used to predict conserved acetylation sites within ERRFI1. PACES employs advanced algorithms and predictive models to analyze conserved acetylation sites across different species [13].

### Data mining

Data on NAT10 and ERRFI1 mRNA expression in CRC tissues and associated clinical information were retrieved from The Cancer Genome Atlas (TCGA) and Gene Expression Omnibus (GEO) databases. For the TCGA analysis, colon cancer data were specifically queried. The Cancer Cell Line Encyclopedia (CCLE), a comprehensive database of various molecular features of cancer cell lines (including gene expression, mutations, and copy number variants [14]), was accessed via the CCLE website (https://portals.broadinstitute.org/ccle) to retrieve relevant gene expression and drug information.

Two gene sets obtained from RNA-seq and RIP-seq were further analyzed for functional annotation of differentially expressed genes (DEGs). Gene Ontology (GO) enrichment analysis (http://www.geneontology.org) and Kyoto Encyclopedia of Genes and Genomes (KEGG) pathway analysis (https://www.genome.jp/kegg/pathway.html) on DEGs were performed. A p-value < 0.05 was used as the cutoff value.

#### Animal studies

Stably transfected cells (1 × 10^7^) were suspended in 200 µL of pre-cooled PBS and injected subcutaneously into the backs of 4- to 6-week-old male BALB/c nude mice (obtained from Liaoning Changsheng Biotechnology Company). The mice were randomly divided into groups, and tumor size and weight were measured throughout the experiment. Tumor volume was calculated using the formula: volume = tumor maximum diameter (L) × right-angle diameter to that axis (W)^2^/2 (mm^3^). All mice were sacrificed at the appropriate time, and the excised tumors were used for IHC staining. All animal experiments adhered to ethical guidelines and were approved by the Medical Ethics Committee of Wuhan University. The mice were housed in appropriate conditions with free access to food and water.

### Synergy determination with SynergyFinder

Approximately 20,000 cells per well were seeded into a 96-well imaging plate and treated with varying concentrations of Remodelin and cetuximab, either alone or in combination, for 24 h. Cell viability was assessed using a CCK-8 assay, and absorbance was measured with a microplate reader (Perkin Elmer, Waltham, Massachusetts, USA). The data were uploaded to SynergyFinder software (https://synergyfinder.fimm.fi) where synergy calculations were performed using the zero-interaction potency (ZIP) method [15]. ZIP synergy scores above 1 indicated synergy (red regions), and scores exceeding 10 indicated strong synergy. A 3D synergy diagram of the drug combination responses was generated to assess the therapeutic efficacy of drug combinations.

#### Mass spectrometry analysis

For mass spectrometry analysis of NAT10 interactions, peptides from SW620 cells transfected with shNAT10 or control vectors were extracted and processed for liquid chromatography-mass spectrometry (LC-MS) analysis by Shanghai OE Biotech Co., Ltd. The methods and details are as previously described [12].

### Statistical analysis

Statistical analysis was carried out with GraphPad Prism 9.0 and SPSS software (V.19.0). For comparison of two groups, *P*-values were computed through a two-sided Student’s t-test. For comparison of more than two groups, *P*-values were calculated using ANOVA. To examine the relationship between NAT10 expression and clinicopathological characteristics, a two-sided X^2^ test was employed. Survival curves were drawn based on the Kaplan-Meier method, and their comparisons were made via the log-rank test. Multivariate survival analysis using Cox proportional hazard regression models were performed to evaluate independent prognostic factors. In all circumstances, statistical significance was defined as a p-value of < 0.05. *P < 0.05, **P < 0.01, ***P < 0.001, ****P < 0.0001.

## Results

### NAT10 acts as a pro-oncogenic factor in CRC cells

**Fig. 1:**
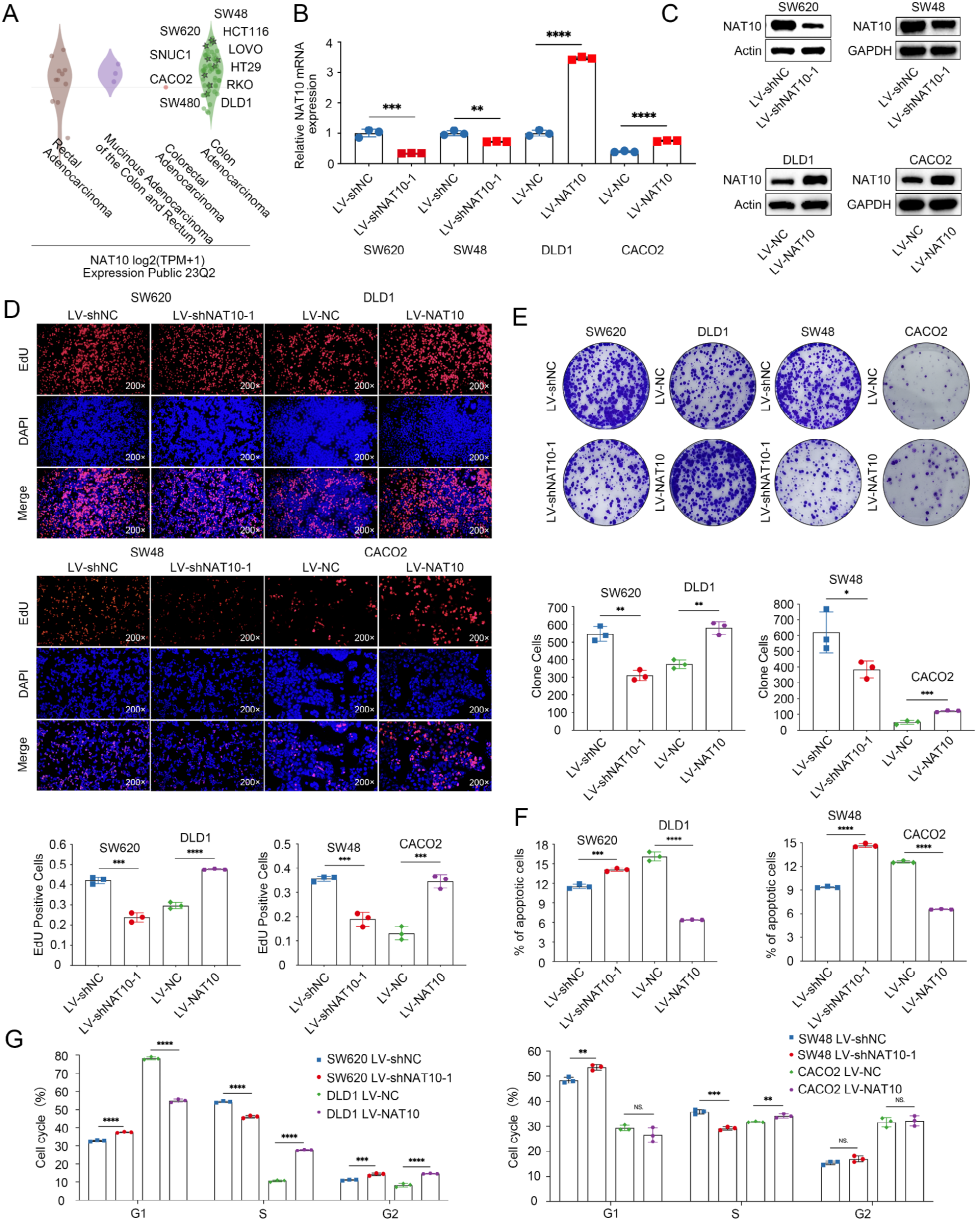
NAT10 promotes proliferation and inhibits apoptosis of CRC cells in vitro. **A**. NAT10 expression in various cell lines based on CCLE datasets. **B**, **C**. Knockdown or over-expression transfection efficiency of NAT10 in SW620, SW48, DLD1 and CACO2 cells determined using RT-qPCR and WB. **D**, **E**. Effects of NAT10 on proliferation were measured using the EdU assays (**D**) and colony formation assays (**E**). **F**. Flow cytometry was used to detect rates of apoptosis (LR + UR) of indicated cells. **G**. The cell cycle distribution was detected by flow cytometry in NAT10 knockdown or overexpression cells. The data are representative of three independent experiments and presented as the mean ± SD. Comparisons were performed using two-tailed unpaired Student’s *t*-tests. *P < 0.05, **P < 0.01, ***P < 0.001, ****P < 0.0001

We first analyzed the relative NAT10 expression levels across different cell lines from the CCLE database. Among CRC cell lines, SW48, SW620, HCT-116, and SNUC1 showed relatively high NAT10 expression, while CACO2, RKO, SW480, and DLD1 showed relatively low NAT10 expression (Fig. 1A). To establish the biological role of NAT10 in CRC cells, we performed knockdown and overexpression assays. We selected two KRAS mutant cell lines (SW620 and DLD1) and two non-KRAS mutant cell lines (SW48 and CACO2) for these assays. Two independent shRNAs targeting NAT10 were used, and their efficacy was confirmed in two independent clones by RT-qPCR and WB (Fig. 1B, C; Supplementary Fig. 1A). EdU assays revealed that NAT10 knockdown by shRNA dramatically slowed the growth and division of SW620 and SW48 cells, whereas NAT10 overexpression in DLD1 and CACO2 cells accelerated growth and division (Fig. 1D, Supplementary Fig. 1B). Colony formation assays demonstrated that NAT10 promoted the long-term growth of CRC cells, conversely, NAT10 knockdown significantly impaired CRC cell proliferation (Fig. 1E, Supplementary Fig. 1C). To further investigate the role of NAT10, we examined its impact on apoptosis in CRC cells. NAT10 knockdown significantly increased the proportion of cells in the apoptosis phase (Fig. 1F, Supplementary Fig. 1D). Cell cycle distribution was also analyzed. NAT10 knockdown arrested the cell cycle of SW620 and SW48 cells in the G1 phase, while NAT10 overexpression in DLD1 and CACO2 cells promoted cell cycle progression (Fig. 1G, Supplementary Fig. 1E). Collectively, these data strongly support a critical role for NAT10 in promoting CRC progression in vitro.

### Remodelin induced reversible growth arrest in CRC cells

**Fig. 2:**
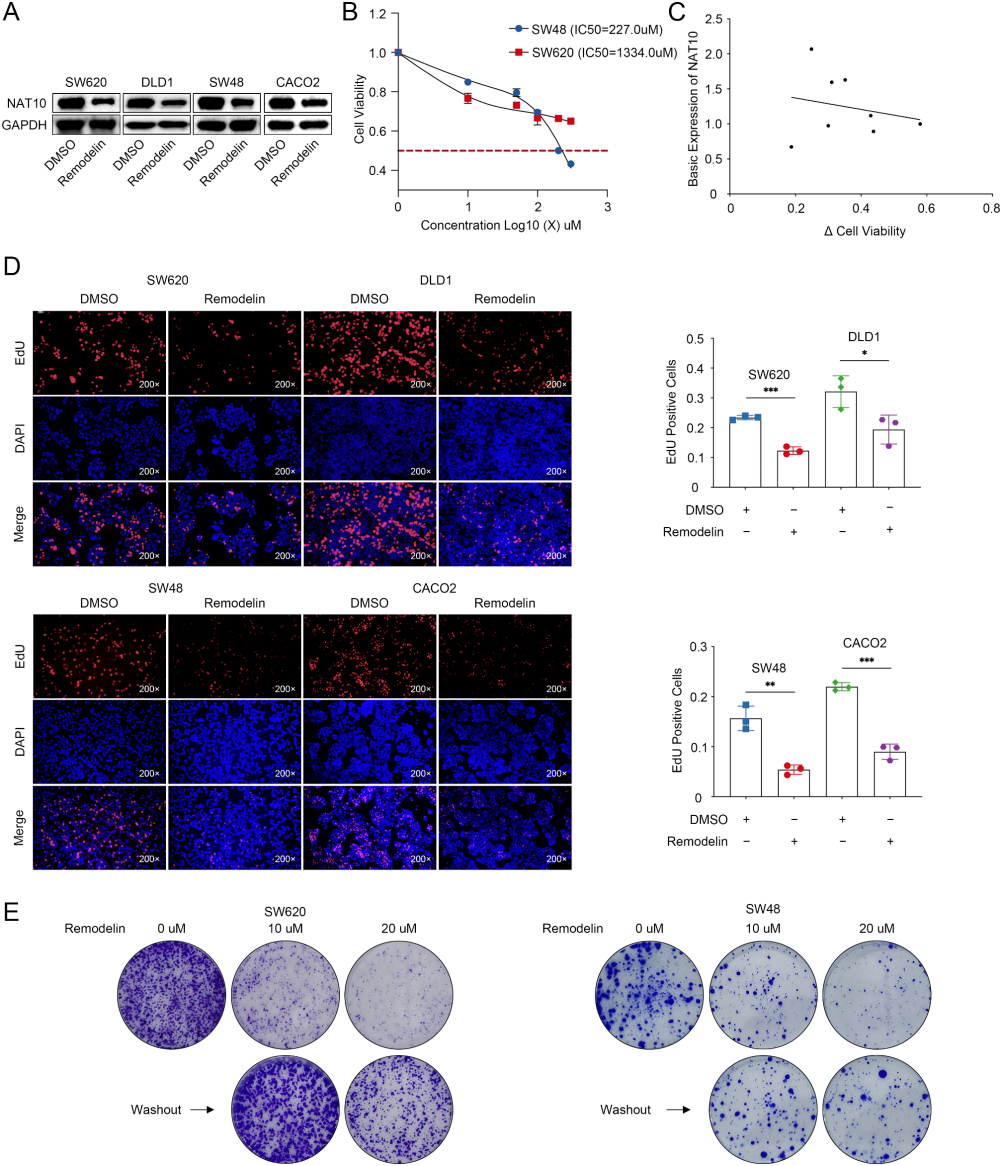
Effects of the NAT10 inhibitor Remodelin on CRC proliferation. **A**. WB analysis of NAT10 expression in a panel of CRC cell lines treated with Remodelin. **B**. CCK-8 assay of SW48 and SW620 cells treated with different concentrations of Remodelin. **C**. The correlation between the basal NAT10 expression content and Remodelin efficacy. **D**, **E**. EdU and colony formation assays were performed to verify the effect of Remodelin on CRC cell proliferation. The data are representative of three independent experiments and presented as the mean ± SD. *P < 0.05, **P < 0.01, ***P < 0.001, ****P < 0.0001, determined using two-tailed unpaired Student’s *t-*tests

Remodelin is an effective small-molecule inhibitor of NAT10 and has been identified as a potential therapeutic strategy for CRC [6]. As expected, Remodelin treatment decreased intracellular NAT10 protein levels in a panel of CRC cell lines (Fig. 2A). Next, we evaluated the anticancer activity of Remodelin using CCK-8 assays. Remodelin inhibited SW620 and SW48 cell growth in a dose-dependent manner (Fig. 2B). However, it remains unknown whether the effect of Remodelin is dependent on NAT10 levels in CRC cell lines. To investigate this, eight cell lines (six KRAS-mutated and two KRAS-wild type) were treated with 20 µM Remodelin for 24 h, and cell viability was assessed by CCK-8 assay. Concurrently, immunoblotting was used to detect the basal protein expression levels of NAT10. Figure 2C shows the correlation between basal NAT10 expression and Remodelin efficacy. We found that the effect of Remodelin is independent of NAT10 levels in CRC cell lines. EdU assays further verified that the proliferative abilities of SW620 and SW48 cells were significantly suppressed by Remodelin (Fig. 2D), suggesting that Remodelin treatment inhibits the proliferation of CRC cells expressing wild-type or mutant KRAS. In further growth assays, as shown in Fig. 2E, after the treatment of SW620 and SW48 cells with Remodelin for 5 days, we observed the prompt resumption of cell growth upon drug withdrawal. This finding indicated that Remodelin induces reversible growth arrest in CRC cells, which might be linked to its limited efficacy.

### Profile of ac4C‑modified genes regulated by NAT10 in CRC cells

**Fig. 3:**
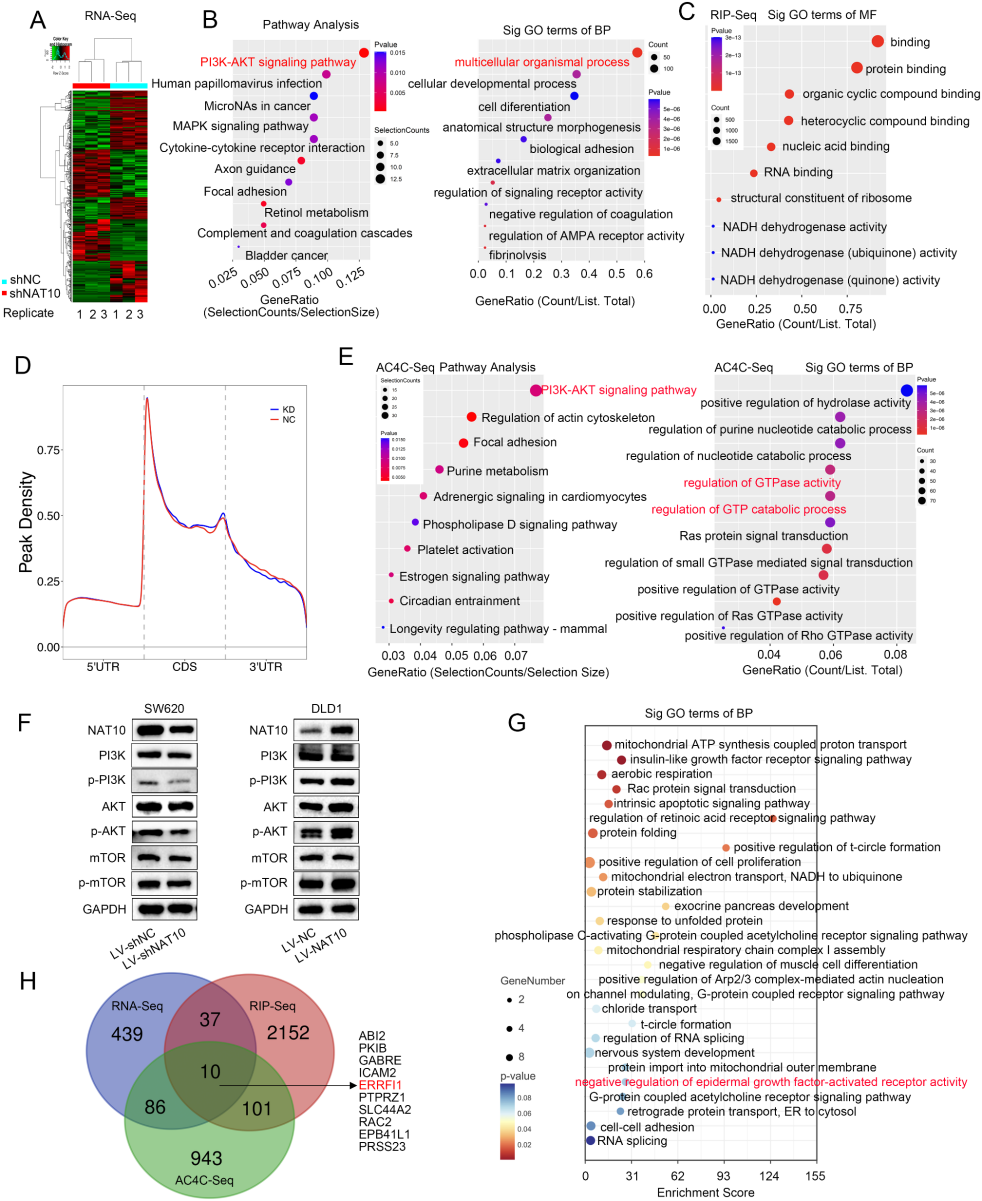
Identification of downstream targets of NAT10 in CRC cells. **A**. Heat map showing differentially expressed genes identified by RNA-seq in NAT10-knockdown cells relative to control cells. Green and red indicate low and high mRNA expression levels, respectively. **B**. Pathway enrichment and GO enrichment analysis of significantly downregulated genes. **C**. GO enrichment analysis of the potential NAT10 binding RNAs. **D**. ac4C peak mainly appeared in coding sequences (CDS) of SW620 cells. **E**. Pathway enrichment and GO enrichment analysis of acRIP-Seq RNA sequence data. **F**. Relative protein levels of PI3K, p-PI3K, AKT, p-AKT, mTOR and p-mTOR in CRC cells following NAT10 knockdown or overexpression. **G**. GO enrichment analysis of genes that overlapped with those identified in RIP-seq and acRIP-seq analysis. **H**. Overlap of genes identified using RNA-seq, RIP-seq, and acRIP-seq

To comprehensively uncover the mechanism by which NAT10 regulates CRC tumorigenesis, we first performed RNA sequencing (RNA-seq) to assess the changes in global gene expression after NAT10 knockdown. NAT10 depletion resulted in the global alteration of 509 genes, including 220 upregulated and 289 downregulated genes (Fig. 3A). A pathway enrichment analysis showed that most of the downregulated genes after NAT10 silencing were involved in the PI3K-AKT signaling pathway, and a Gene Ontology (GO) functional enrichment analysis indicated that NAT10 might be associated with multicellular organismal processes (Fig. 3B). NAT10, as an ac4C ‘writer’ protein, mainly participates in epigenetic regulation by binding and affecting ac4C acetylated transcripts and has crucial effects on mRNA metabolism [16]. We performed RIP-seq and acRIP-seq using SW620 NAT10-knockdown cells. As expected, a GO analysis revealed that most of the potential NAT10 binding RNAs function in protein binding (Fig. 3 C). After analyzing the distribution on mRNA, we detected ac4C peaks mainly in coding sequences (CDS), consistent with previously reported data (Fig. 3D). Importantly, in line with our RNA-seq results, ac4C-seq revealed that NAT10-mediated ac4C-modified genes were also involved in the PI3K-AKT signaling pathway (Fig. 3E). Therefore, we evaluated the effect of NAT10 on PI3K-AKT pathway using WB, revealing that silencing NAT10 decreased PI3K, AKT and mTOR phosphorylation levels (Fig. 3F). However, a GO enrichment analysis of the genes that overlapped with those identified by RIP-seq and acRIP-seq also showed that the NAT10-binding and ac4C-modified genes were involved in the negative regulation of epidermal growth factor-activated receptor activity (Fig. 3G). We then integrated RNA-seq, acRIP-seq, and RIP-seq to identify potential NAT10-regulated ac4C targets. As shown in a Venn diagram, ten potential NAT10-binding RNAs displayed significant dysregulation at the mRNA and ac4C levels (Fig. 3H). These 10 genes included *ERRFI1*, a feedback inhibitor of both EGFR and ERBB2. ERRFI1 functions as a regulator of EGFR-mediated signaling and is involved in tumor development and chemoresistance in CRC [8, 9]. We hypothesized that ERRFI1-mediated EGFR signaling activation contributes to the reversible growth arrest induced by Remodelin and its limited efficacy.

### ERRFI1 was regulated by NAT10 and participated in CRC malignant progression

**Fig. 4:**
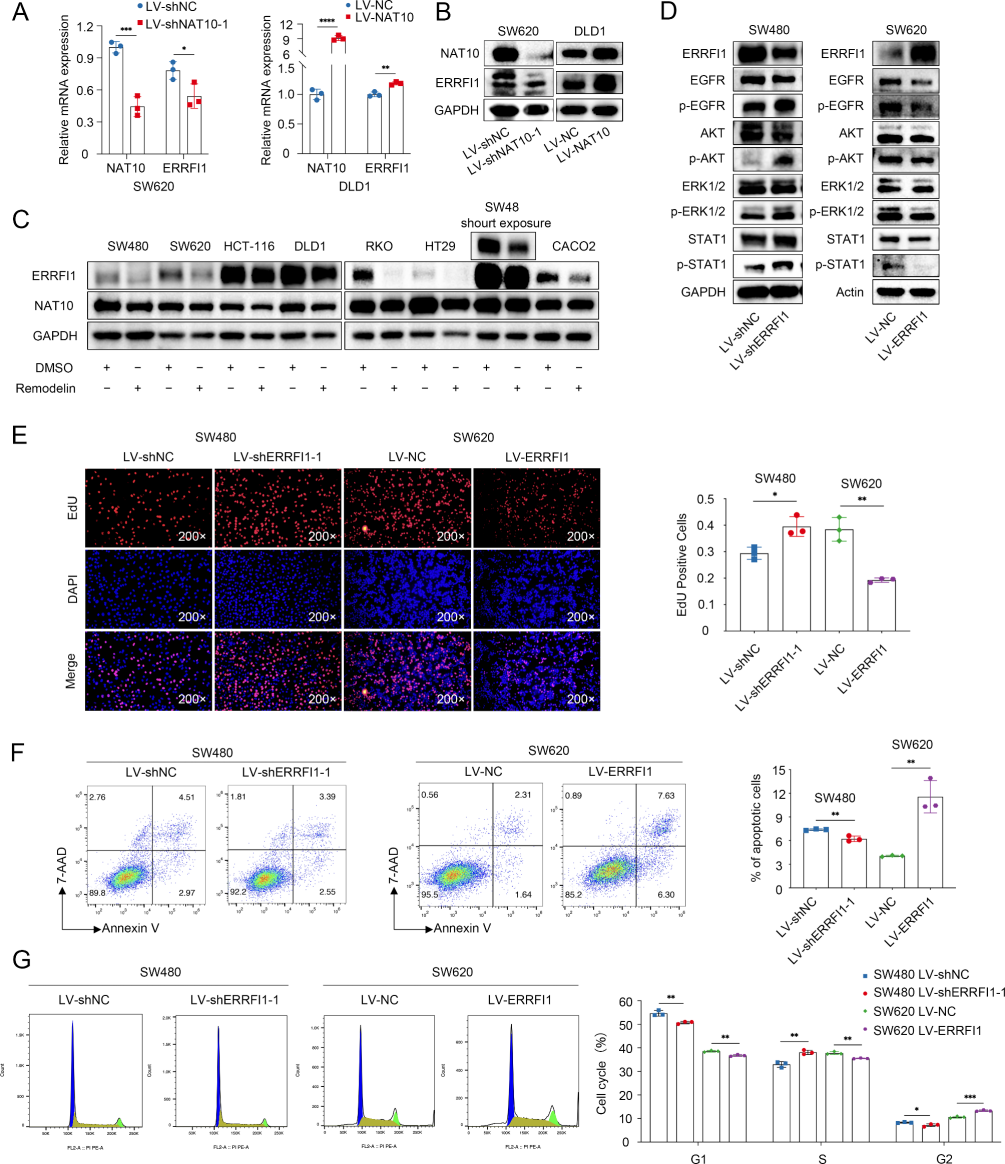
ERRFI1 was regulated by NAT10 and act as a critical role in CRC malignant progression. **A**. RT-qPCR analysis of NAT10 and ERRFI1 expression in CRC cell lines. **B**. NAT10 and ERRFI1 were detected by WB in the indicated cells. **C**. WB analysis of NAT10 and ERRFI1 expression in a panel of CRC cell lines treated with Remodelin (20 µM). **D**. Protein expression and phosphorylation levels of EGFR, AKT, ERK, and STAT1 in the indicated cells were measured by WB. **E**. The effects of ERRFI1 on CRC cell proliferation were measured using an EdU assay. **F**. Flow cytometry was performed to detect the apoptotic rate (LR + UR) of the indicated cells. **G**. Cell cycle distribution was detected using flow cytometry in ERRFI1 knockdown or overexpression cells. The data are representative of three independent experiments and presented as the mean ± SD. *P < 0.05, **P < 0.01, ***P < 0.001, ****P < 0.0001, as determined using two-tailed unpaired Student’s *t*-tests

We investigated ERRFI1 expression via RT-qPCR and WB after NAT10 knockdown and overexpression. As expected, ERRFI1 expression at the mRNA and protein levels decreased after NAT10 knockdown by shRNA and was higher in NAT10 overexpression cells than in wild-type cells (Fig. 4A, B). In addition, we observed a significant decline of NAT10 and ERRFI1 protein expression after the application of Remodelin in 8 chosen cell lines (Fig. 4 C). Previous studies have demonstrated the suppressive role of ERRFI1 in cell proliferation. However, ERRFI1 inactivation contributes to the acquired resistance to targeted therapy with EGFR tyrosine kinase inhibitors in cancer cells. We evaluated the effects of ERRFI1 upregulation in SW480 and downregulation in SW620 cells on the EGFR signaling pathway. The results showed that the phosphorylation levels of EGFR, AKT, ERK, and STAT1 increased in ERRFI1-knockdown cells and decreased in ERFFI1 overexpression cells (Fig. 4D). An EdU assay further showed that ERRFI1 knockdown promoted the growth and division of SW480 cells, whereas ERRFI1 overexpression in SW620 cells showed the opposite effects (Fig. 4E, Supplementary Fig. 2A, B). Additionally, ERRFI1 overexpression increased the proportion of apoptotic cells and decreased the proportion of cells in the S - phase, whereas ERRFI1 knockdown in SW480 cells inhibited apoptosis and promoted cell cycle progression (Fig. 4F, G, Supplementary Fig. 2C, D). Collectively, these data provided evidence that ERRFI1 is regulated by NAT10 and plays a critical inhibitory role in the malignant progression of CRC.

### NAT10‑mediated ac4C modification maintains *ERRFI1* stability

**Fig. 5:**
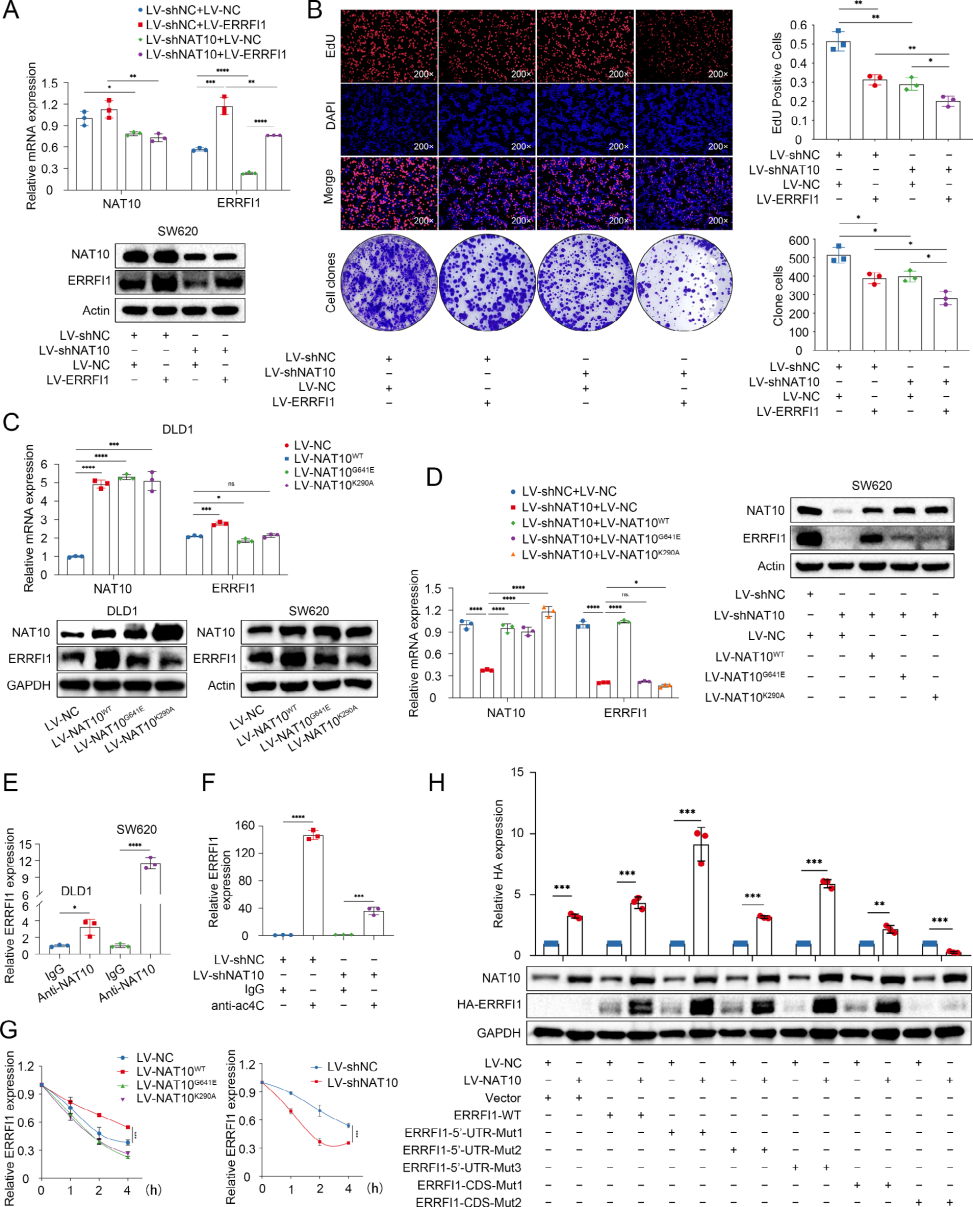
NAT10‑mediated ac4C modification maintains *ERRFI1* transcript stability. **A**. RT-qPCR and WB analysis of NAT10 and ERRFI1 expression in NAT10-ERRFI1 rescued cell lines. **B**. EdU and colony formation assay of NAT10-ERRFI1 rescued cell lines. **C**, **D**. RT-qPCR and WB analysis of NAT10 and ERRFI1 expression in the indicated CRC cell lines. **E**. RIP assays with anti-NAT10 and anti-IgG antibodies were performed to analyze the relative NAT10 enrichment in *ERRFI1* mRNA. **F**. Relative ac4C levels of *ERRFI1* transcripts were evaluated in the indicated cells using acRIP-qPCR. **G**. *ERRFI1* mRNA stability in cells treated with 5 µg/mL actinomycin D (ACTD). **H**. RT-qPCR and WB analysis of NAT10 and HA-ERRFI1 expression in the indicated CRC cell lines. The data are representative of three independent experiments and presented as the mean ± SD. *P < 0.05, **P < 0.01, ***P < 0.001, ****P < 0.0001, as determined using two-tailed unpaired Student’s t-tests

To generate rescue cell lines, SW620 cells were transfected with lentiviruses carrying sh-NAT10 and/or ERRFI1. Transfection efficiency was verified by RT-qPCR and WB assays (Fig. 5A). The proliferation assay showed that ERRFI1 overexpression potentiated the effect of NAT10 knockdown on CRC cell proliferation (Fig. 5B). To test whether NAT10 regulation of ERRFI1 was dependent on its ac4C activity, wild-type NAT10 or mutant NAT10 lacking a functional acetyltransferase domain (G641E) or RNA helicase domain (K290A) was introduced into DLD1 and SW620 cells. As shown in Fig. 5C, overexpression of wild-type NAT10, but not the K290A or G641E mutants, enhanced ERRFI1 expression, indicating that NAT10 mainly affects ERRFI1 expression via its RNA acetylation activity. We then re-expressed wild-type NAT10 or mutant NAT10 in NAT10-knockdown cells and found that wild-type NAT10, but not NAT10 mutants, rescued the expression of ERRRFI1, further indicating that the function of NAT10 in ac4C modification is indispensable for its role in mediating ERRFI1 expression (Fig. 5D). Furthermore, the interaction between NAT10 and *ERRFI1* mRNA was verified through RIP followed by RT–qPCR assays, which showed that NAT10 was significantly enriched in *ERRFI1* mRNA (Fig. 5E). Consistent with the acRIP-seq results, acRIP confirmed the abundance of ac4C modifications in *ERRFI1* mRNA (Fig. 5F). To this end, we immediately tested whether NAT10 regulate the stability of *ERRFI1* mRNA. We therefore treated CRC cells with actinomycin D (5 µg/mL) to examine RNA decay after NAT10 knockdown or overexpression. The results indicated that NAT10 enhanced the stability of *ERRFI1* mRNA in an ac4C-dependent manner (Fig. 5G).

To identify the key ac4C sites of *ERRFI1* modified by NAT10, we further analyzed the acetylation peaks of *ERRFI1* mRNA. acRIP-seq data showed that the ac4C peaks were distributed in the 5’-untranslated regions (5’-UTR) and CDS region of *ERRFI1* mRNA (txStart-txEnd: chr1: 8015494–8015692). Meanwhile, the PACES tool predicted the potential acetylation site was on the region of CDS (Supplementary Fig. 3A). Accordingly, we constructed one wild-type and five mutant HA-labeled *ERRFI1* plasmids (Supplementary Fig. 3B). Cytosine (C) was replaced with thymine (T) in the potential ac4C peak of the mutant *ERRFI1* 5’-UTR or CDS region. We then transfected these plasmids into DLD1-LV-NC/LV-NAT10 cells. The RT-qPCR and WB analysis showed that NAT10 overexpression failed to enhance ERRFI1 mRNA and protein expression only in the CDS-Mut2 mutant group, suggesting that the ac4C sites of may be located on the *ERRFI1* mRNA CDS region (Fig. 5H).

### EGFR inhibition improves the therapeutic efficacy of remodelin in vivo and in vitro

**Fig. 6:**
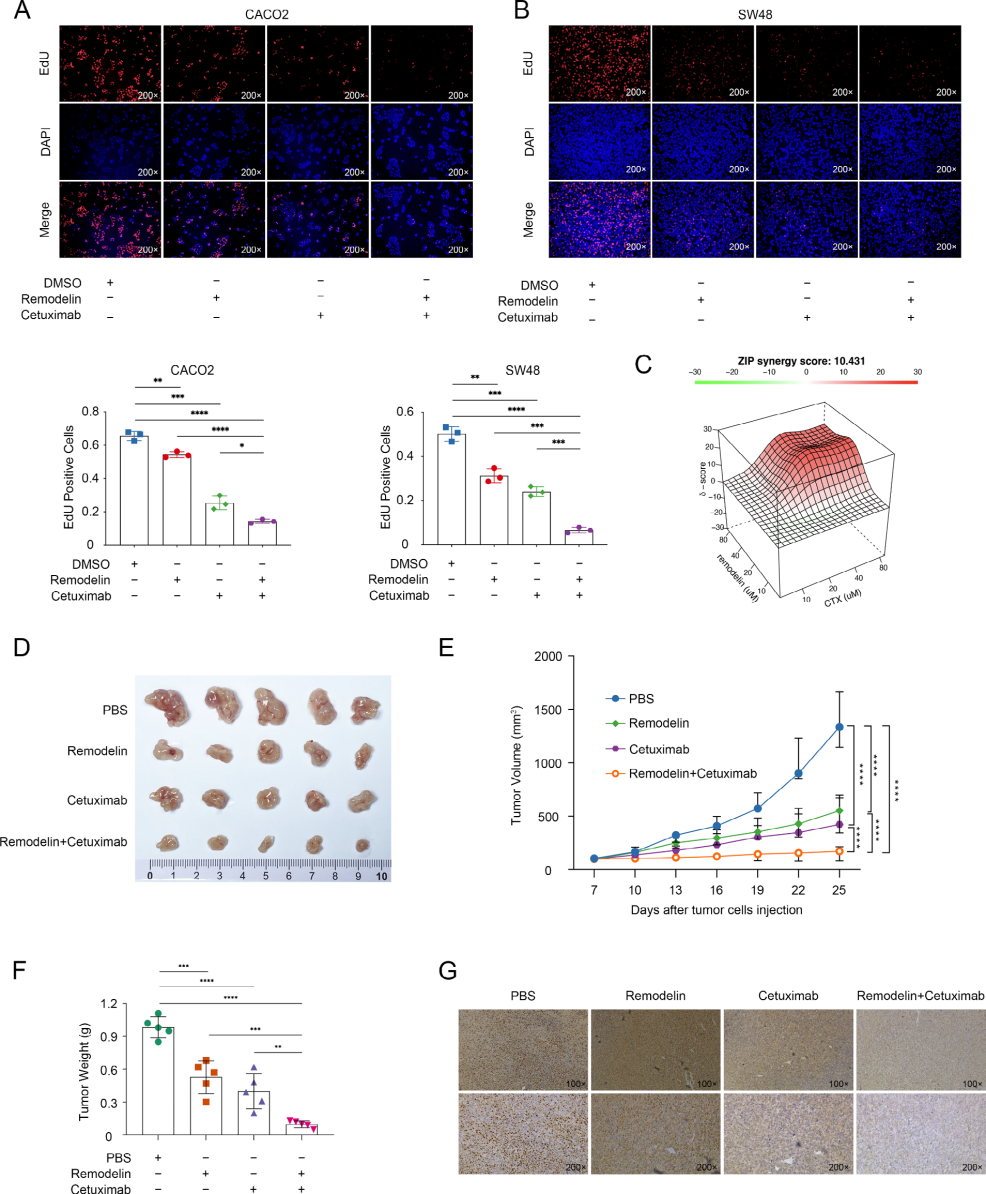
Dual targeting of NAT10 and EGFR effectively inhibited the proliferation of wild-type KRAS CRC cells. **A**, **B**. EdU assays were used to verify the cooperative effects of Remodelin and cetuximab in KRAS wild-type CRC cells. **C**. Synergy diagram of Remodelin and cetuximab, calculated with the online SynergyFinder software. SW48 cells were treated with different concentrations of reagents at various concentrations (Remodelin: 0, 10, 20, 40, 80 µM; cetuximab: 0, 10, 20, 40, 80 µM) for 24 h. Relative cell viability was subsequently measured. ZIP values were simulated using zero interaction potency model analysis. **D**. Representative images of subcutaneous tumors in the indicated groups. **E**. Tumor volumes were measured every 3 days. **F**. Tumor weights are expressed as the mean ± SD of five mice. **G**. Representative images of IHC staining with an anti-Ki67 antibody in tumors formed in the indicated groups. The data are representative of three independent experiments and presented as the mean ± SD. *P < 0.05, **P < 0.01, ***P < 0.001, ****P < 0.0001, calculated using two-tailed unpaired Student’s *t*-tests

Given that NAT10 inhibition resulted in the downregulation of ERRFI1, which could reactivate EGFR signaling in CRC cells, we further evaluated whether the EGFR-specific monoclonal antibody cetuximab can inhibit the reactivation of EGFR signaling and improve the therapeutic efficacy of Remodelin in KRAS wild-type CRC cells. The combined effects of Remodelin and EGFR inhibitors were observed in EdU assays (Fig. 6A, B), indicating that EGFR inhibition effectively augmented the therapeutic efficacy of Remodelin in vitro. We further examined synergistic effects of EGFR inhibitors and Remodelin using a short-term proliferation assay. We detected synergistic effects for each drug pair, with zero-interaction potency (ZIP) synergy scores ranging from 15 to 30 (where a score greater than 1 indicates a synergistic interaction) (Fig. 6 C). We tested the combined effects of Remodelin and EGFR inhibitors in a KRAS wild-type CRC cell xenograft mouse model. Mice bearing SW48 xenografts were treated with vehicle, Remodelin, cetuximab, or a combination of Remodelin and cetuximab for 18 days. Remodelin alone did not suppress xenograft tumor growth substantially within the treatment period, the combination resulted in greater tumor shrinkage in vivo, demonstrating robust and synergistic antitumor activity, suggesting a sensitizing effect of cetuximab on Remodelin (Fig. 6D–F). Importantly, the addition of cetuximab to Remodelin was well tolerated, and no significant adverse effects, such as weight loss or treatment-related mortality, were observed. An IHC analysis demonstrated that cotreatment with Remodelin and cetuximab reduced Ki67 expression significantly in SW48 xenograft tumors (Fig. 6G). Collectively, these in vivo data suggest that the combination of Remodelin and EGFR inhibitors has potent antitumor activity.

### Anticancer effect of the combination of 5-Fu, remodelin and EGFR inhibition in KRAS wild-type CRC

**Fig. 7:**
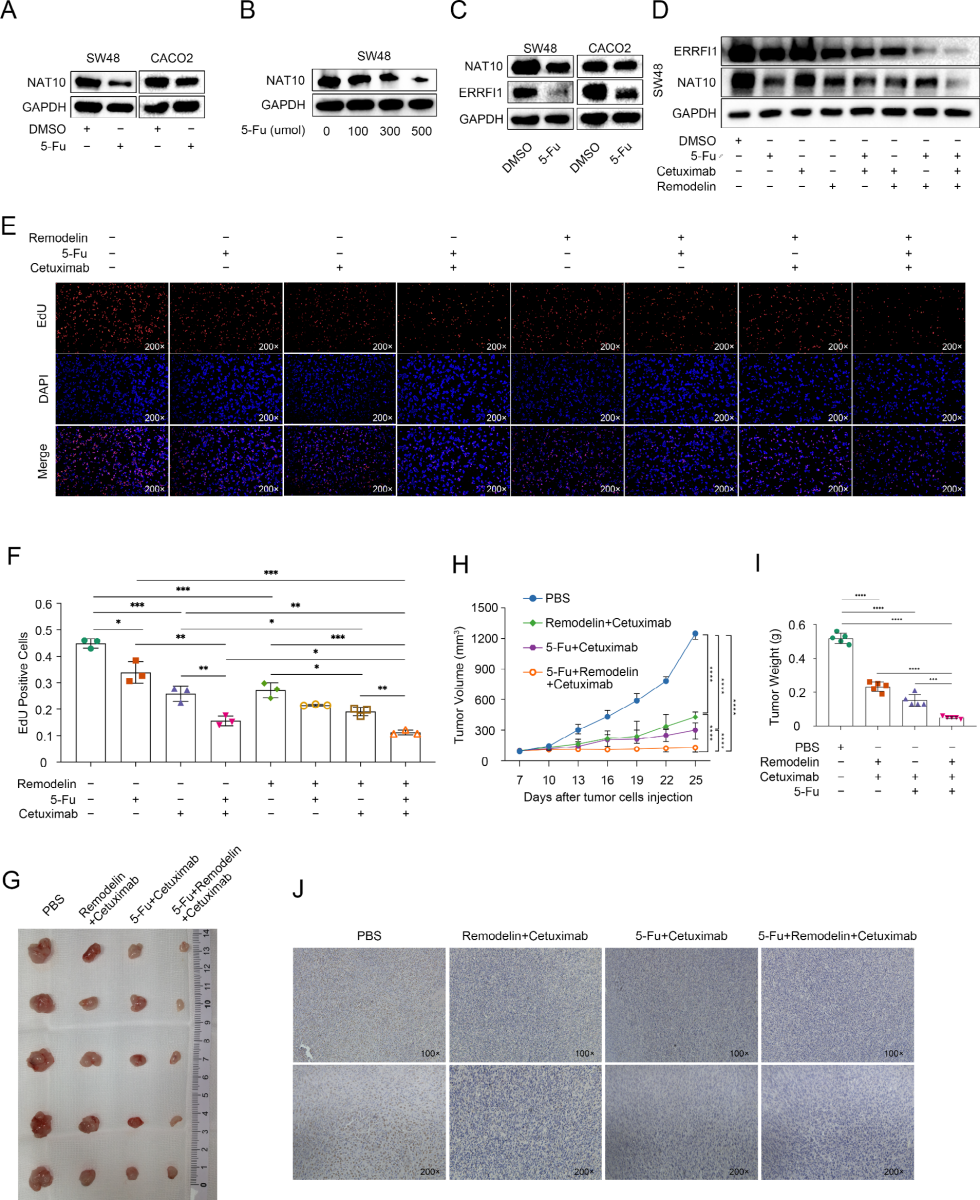
Triple combination therapy with 5-Fu, Remodelin, and EGFR inhibition showed a striking anticancer effect in KRAS wild-type CRC. **A**-**B**. WB analysis of NAT10 protein expression in CRC cell lines. **C**. WB analysis of NAT10 and ERRFI1 expression in CRC cell lines. D. WB analysis of NAT10 and ERRFI1 expression in SW48 cells treated with indicated drugs. **E**, **F**. EdU assays was used to verify the effect of the triple combination therapy on the proliferation of CRC cells. **G**. Representative images of subcutaneous tumors in the indicated groups. **H**. Tumor volumes were measured every 3 days. **I**. Tumor weights are expressed as the mean ± SD of 5 mice. **J**. Representative images of IHC staining with an anti-Ki67 antibody in tumors formed in the indicated group. The data are representative of three independent experiments and presented as the mean ± SD. *P < 0.05, **P < 0.01, ***P < 0.001, ****P < 0.0001, using two-tailed unpaired Student’s *t*-tests

Since ERRFI1 is a driver of 5-FU resistance in CRC [17] and the NAT10-mediated epigenetic modification of mRNA is reversible and responds rapidly to cellular stress, we evaluated whether 5-FU treatment affects the NAT10-ERRFI1 axis. In a panel of CRC cells, NAT10 expression was significantly downregulated following 5-Fu treatment (Fig. 7A, Supplementary Fig. 4A). Moreover, treatment with increasing concentrations of 5-Fu for 24 h resulted in a dose-dependent decrease in NAT10 expression in both DLD1 and SW48 cells (Fig. 7B, Supplementary Fig. 4B), suggesting that 5-Fu has an inhibitory effect on NAT10 expression in CRC cells. Furthermore, NAT10 and ERRFI1 protein levels were consistently reduced by 5-Fu treatment (Fig. 7 C, Supplementary Fig. 4C). A WB assay showed that 5-Fu did not decrease ERRFI1 protein levels when NAT10 was overexpressed (Supplementary Fig. 4D). Collectively, these data indicated that the 5-Fu-induced NAT10-ERRFI1 reduction may result in EGFR signaling reactivation, which emphasizes the necessity for the combination of 5-Fu and cetuximab, as recommended by clinical guidelines. However, patients who benefit from 5-Fu and cetuximab treatment are restricted to a population diagnosed with left colon cancer [18]. The effectiveness of this combined treatment strategy in many patients remains limited, and novel targets are needed to improve efficiency. We then tested the effect of combining 5-Fu, cetuximab and Remodelin on NAT10 and its target in SW620 and SW48 cells. As shown in Fig. 7D and supplementary Fig. 4E, both 5-Fu and Remodelin treatment can lead to a decline in NAT10 and ERRFI1 expression, and the combination of 5-Fu and Remodelin leads to a more significant decline in NAT10 and ERRFI1 expression, while cetuximab has little effect on NAT10 and ERRFI1 protein expression. We next tested the therapeutic efficacy of the combination of 5-Fu, cetuximab, and Remodelin. EdU assay results showed that the anticancer activity in the 5-Fu, cetuximab, and Remodelin combination treatment group was significantly greater than that in the control groups (Fig. 7E, F). We then tested the combined effects of 5-Fu, cetuximab, and Remodelin in a KRAS wild-type CRC cell xenograft mouse model. As expected, triple-combination therapy significantly suppressed tumor growth (Fig. 7H-I). Importantly, none of these treatments showed overt systemic toxicity or a significant effect on body weight. IHC analysis demonstrated that triple-combination therapy significantly reduced Ki67 expression in SW48 xenograft tumors (Fig. 7J). Collectively, these data suggest that simultaneous targeting of NAT10 may further improve the efficacy of 5-Fu and cetuximab treatment in patients with CRC.

### 5-Fu destabilized NAT10 by promoting UBR5-meidated ubiquitination of NAT10

**Fig. 8:**
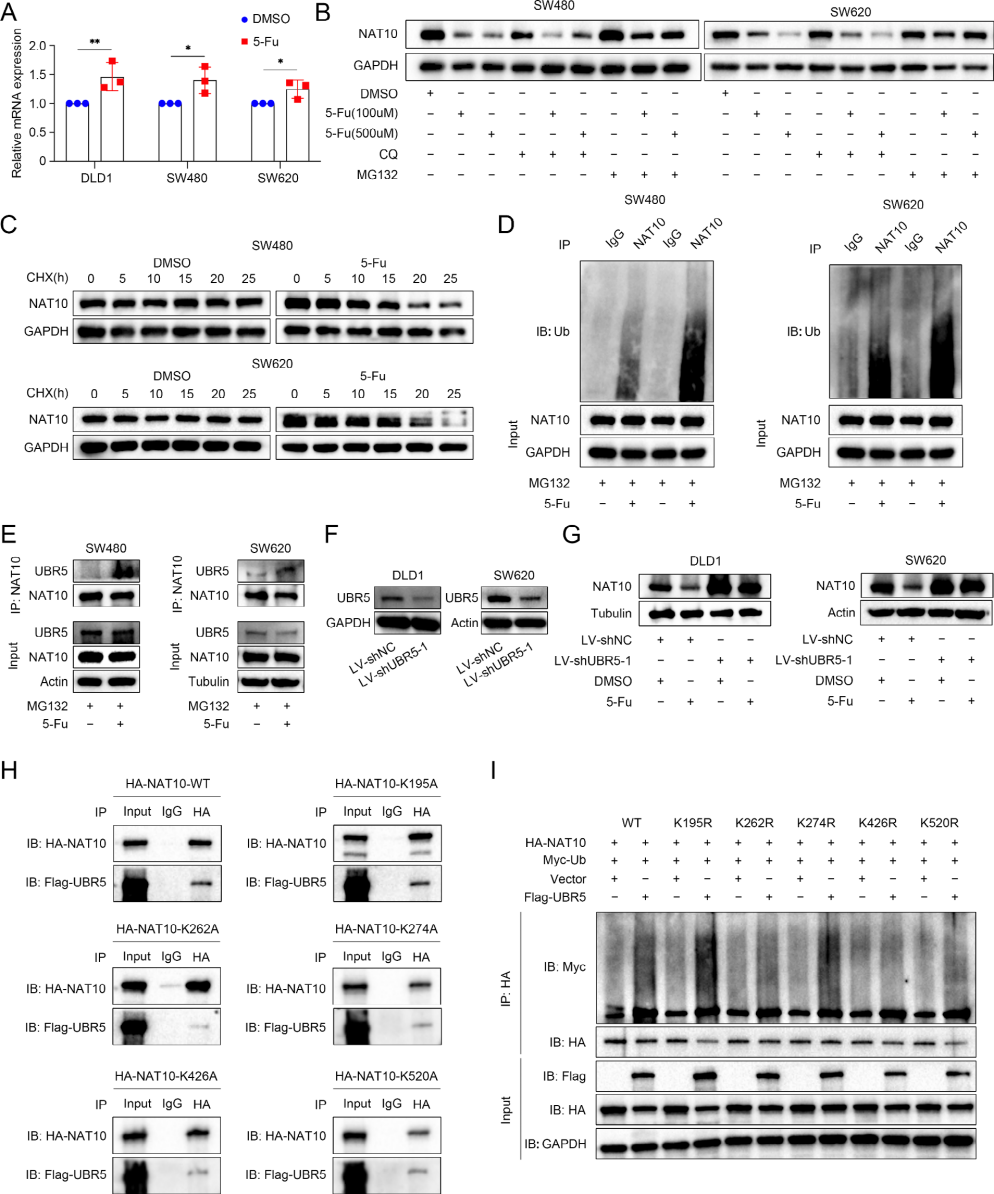
NAT10 interacts with UBR5 and is ubiquitinated by ubiquitin ligase. **A**. RT-qPCR analysis of *NAT10* expression in CRC cell lines. **B**. CQ (20 µM) was used to inhibit lysosomal degradation and MG132 (100 nM) was employed to repress proteasomal degradation in SW620 cells. **C**. Half-life analysis of NAT10 in SW480 and SW620 cells treated with 50 µg/mL cycloheximide (CHX) for the indicated times. **D**. Ubiquitination assay was performed on the indicated cells. **E**. Co-IP was performed to determine the relationship between NAT10 and UBR5 expression. **F**. Knockdown of UBR5 in DLD1 and SW620 cells was confirmed by WB. **G**. WB analysis of NAT10 in indicated cells treated or not treated with 5-Fu (500 µM). H, I. HA-NAT10 (WT or indicated K-R mutants) was co-expressed with MYC-Ub and Flag-UBR5 plasmids. After MG132 (100 nM, 8 h) treatment, IP was performed with HA antibody, followed by immunoblotting with indicated antibodies. The data are representative of three independent experiments and presented as the mean ± SD. *P < 0.05, **P < 0.01, ***P < 0.001, ****P < 0.0001, using two-tailed unpaired Student’s *t*-tests

We explored how 5-Fu downregulated NAT10 expression in CRC cells. Given that 5-Fu did not decrease the mRNA level of *NAT10* (Fig. 8A), we hypothesized that 5-Fu may regulate NAT10 at the post-transcriptional level. Therefore, we tested whether 5-Fu affects NAT10 protein degradation. As shown in Fig. 8B, the proteasome inhibitor MG132, but not the autophagy inhibitor chloroquine (CQ), which abolishes lysosomal protein degradation, led to the accumulation of endogenous NAT10 in CRC cells upon 5-Fu treatment, suggesting that 5-Fu promotes the proteasome-dependent degradation of NAT10 in CRC cells. Moreover, 5-Fu treatment reduced the half-life of NAT10 after treatment with the protein synthesis inhibitor cycloheximide (CHX) (Fig. 8 C). The ubiquitination levels of NAT10 increased dramatically in 5-Fu treated cells (Fig. 8D). To further explore the molecular mechanism by which 5-Fu downregulated NAT10, we analyzed NAT10-interacting proteins identified by mass spectrometry of NAT10-specific complexes from SW620 cells (Supplementary Fig. 4F). Among the NAT10-binding proteins, UBR5 (Ubiquitin protein ligase E3 component n-recognin 5) was of special interest, as it is homologous to an E6AP C-terminus (HECT) domain-containing ubiquitin ligase, which plays a critical role in cancer progression [19]. The endogenous NAT10-UBR5 interaction was confirmed by immunoprecipitation experiments, and 5-Fu treatment strengthened the interaction between NAT10 and UBR5 (Fig. 8E). We then verified that whether 5-Fu promotes NAT10 degradation in a UBR5-dependent manner by first knocking down UBR5 in DLD1 and SW620 cells (Fig. 8F, Supplementary Fig. 4G, H), followed by treatment with 5-Fu. As shown in Fig. 8G and Supplementary Fig. 4I, J, 5-Fu failed to reduce NAT10 protein levels after UBR5 knockdown, suggesting that UBR5 mediates NAT10 degradation upon 5-Fu treatment.

To identify the NAT10 site(s) ubiquitinated by UBR5, we employed mass spectrometry to examine all possible Lys ubiquitination sites in NAT10 using SW620 cells. Using this approach, we found that the lysine K989, K266, K520, K256, K274, K195, K689, K61, K802, K447, K426 and K262 residues were conjugation sites that might underwent UBR5-mediated ubiquitination (Supplementary Table 4). Among the identified sites, five sites (K195, K262, K274, K426 and K520) were found to be highly conserved in different species (Supplementary Fig. 5). Therefore, we then mutated these lysine residues to arginine (K195R, K262R, K274R, K426R and K520R) and performed ubiquitination assay to examine which lysine is required for UBR5-mediated NAT10 ubiquitination. Of note, none of the NAT10 mutants affected its ability to interact with UBR5 (Fig. 8H). However, we found that UBR5 overexpression in 293T cells obviously elevated the ubiquitination level of wild-type NAT10. This increase in NAT10 ubiquitination could also be observed in K195R, K262R, K274R and K520R mutants of HA-NAT10, but not in K426R mutant (Fig. 8I), indicating that the K426 residue of NAT10 preferred to be the ubiquitination site for UBR5.

### NAT10 is upregulated in CRC cells and tissues

**Fig. 9:**
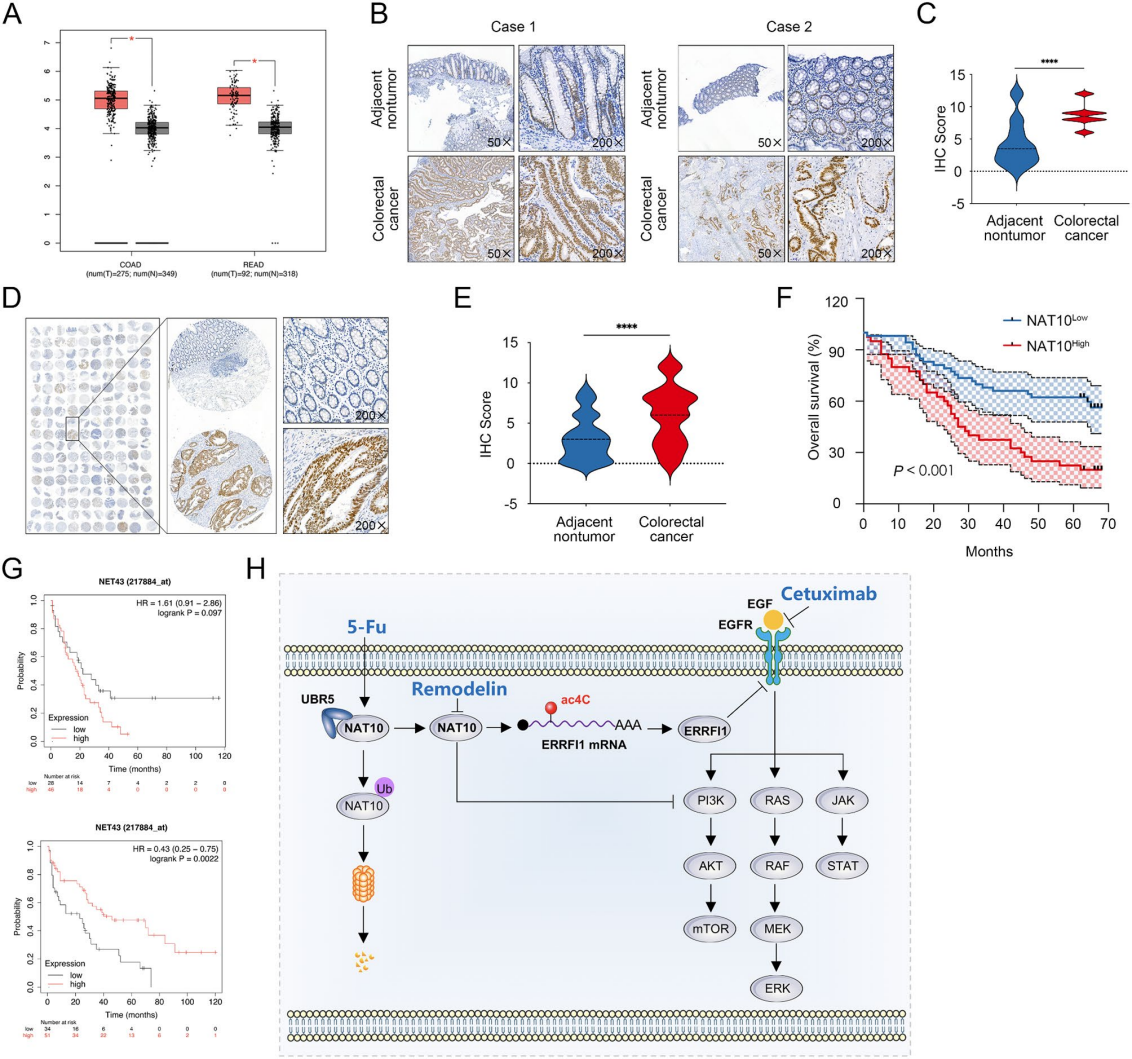
Upregulation of NAT10 in CRC cells and a schematic model of the UBR5-NAT10-ERRFI1 axis. **A**. NAT10 expression profiles across colon adenocarcinoma (COAD) and rectal adenocarcinoma (READ) tumor samples (in red) and paired normal tissues (in black) based on the GEPIA data resource. **B**, **C**. Protein levels of NAT10 determined by IHC in 20 matched CRC tumors and adjacent tissues and quantitative analysis (*n* = 20). **D**. Protein levels of NAT10 were detected by IHC and a tissue microarray for 90 patients with CRC. Representative images of the tumor and paired adjacent normal tissues stained with NAT10 are shown. **E**. Quantitative analysis of IHC staining of NAT10 expression in TMA (*n* = 90). **F**. CRC samples were divided into NAT10-high and NAT10-low expression groups according to the median expression of NAT10; Kaplan–Meier survival analysis showed that the upregulation of NAT10 was significantly associated with a poor overall survival rate. **G**. Kaplan–Meier analysisof PPS for patients with CRC based on NAT10 expression using the online tool Kaplan–Meier Plotter (Up: KRAS mutated type; Down: KRAS wild type). **H**. Schematic of the UBR5-NAT10-ERRFI1 axis. The data are representative of three independent experiments and presented as the mean ± SD. *P < 0.05, ****P < 0.0001, determined using two-tailed unpaired Student’s *t*-tests

We evaluated whether our findings could be extended to patients with CRC. We first analyzed *NAT10* expression at the mRNA level in colon adenocarcinoma and rectal adenocarcinoma samples and paired non-malignant tissues based on GEPIA data. Tumor tissues had higher *NAT10* expression levels than those of non-tumor tissues (Fig. 9A). An IHC analysis was performed with a cohort of 20 CRC specimen pairs (primary tumor and corresponding normal tissues) acquired from the Xijing Hospital of Digestive Diseases. We found that NAT10 was significantly overexpressed in primary CRC tissues (Fig. 9B and C). Next, a tissue microarray (TMA), including 90 patient samples, was prepared to further explore NAT10 protein levels in CRC. As shown in Fig. 9D and E, NAT10 was predominantly localized in the nuclei of CRC cells, and higher NAT10 expression in CRC tissues than in control tissues was reflected by the higher degree of staining and higher H-scores. Moreover, we validated the prognostic value of NAT10 at the protein level, revealing that the survival rate was lower in patients with high NAT10 expression than in those with low NAT10 expression (Fig. 9F). Similarly, a survival analysis based on a larger clinical sample from the Kaplan–Meier Plotter online tool revealed that in patients harboring mutant KRAS, a higher expression level of NAT10 is associated with a worse prognosis; however, in patients with wild-type KRAS, high expression levels of NAT10 are associated with a favorable prognosis. Therefore, we infer that EGFR inactivation mediated by NAT10-ERRFI1 may contribute to a better prognosis in patients with CRC with wild-type KRAS. Taken together, these results reveal that the ac4C writer NAT10 is upregulated in CRC and is significantly associated with the prognosis of patients with CRC.

## Discussion

Treatment options for patients with advanced CRC are limited. Consequently, a better understanding of the pathogenic mechanisms and identification of novel therapeutic targets are urgently needed to develop effective treatment strategies. In this study, we comprehensively evaluated the role of NAT10 and the therapeutic effects of the NAT10 inhibitor Remodelin in CRC by integrating transcriptomic, genetic, and epigenetic analysis using clinical samples, cell lines, and xenograft mouse models. We found that NAT10 plays a pro-oncogenic role in CRC cells, consistent with previous findings. Notably, the treatment of CRC cells with Remodelin induced reversible growth arrest and demonstrated limited efficacy. Mechanistically, we observed that ERRFI1 was downregulated in an ac4C-dependent manner following NAT10 knockdown, which then activated the EGFR pathway, thereby counteracting CRC inhibition. Consequently, we combined Remodelin with the EGFR-specific monoclonal antibody cetuximab and found that EGFR inhibition effectively enhanced the therapeutic efficacy of Remodelin in vitro and in vivo. Furthermore, 5-Fu treatment enhanced the interaction between NAT10 and UBR5, increasing the ubiquitin-mediated degradation of NAT10. Thus, 5-Fu treatment may lead to EGFR reactivation in a NAT10-ERRFI1 axis-dependent manner. We verified that Remodelin improves the efficacy of chemotherapy-based cetuximab treatments in vitro and in vivo. In summary, our study presents a promising treatment strategy for patients with wild-type KRAS/NRAS/BRAF colorectal tumors.

NAT10, the only enzyme known to catalyze ac4C RNA modification, regulates gene expression by modulating RNA stability and translation efficiency [16, 20]. Mounting evidence suggests that aberrant regulation of NAT10 and ac4C RNA modification is associated with tumor progression [5]. In CRC, NAT10 was identified as an oncogene, regulating the Wnt/β-catenin signaling pathway to promote CRC progression via ac4C acetylation of *KIF23* mRNA [6]. Ferroptosis suppressor protein 1 (*FSP1*) mRNA is modified by NAT10, and this epigenetic modification is associated with the inhibition of ferroptosis in CRC cells [21]. Moreover, the prognostic value of NAT10 overexpression has been documented [6]. These studies demonstrate that NAT10 is a potential target for inhibiting the malignant progression of CRC. Consistent with previous reports, we verified that NAT10 is highly expressed in CRC and promotes CRC proliferation. However, the NAT10-specific small-molecule inhibitor Remodelin induced reversible growth arrest in CRC cells and showed limited efficacy. To address this, we integrated RNA-Seq, acRIPseq, RIP-Seq, and molecular experiments and found that NAT10 inhibition promotes EGFR reactivation by downregulating ERRFI1, a crucial inhibitor of EGFR. Based on this EGFR feedback model, we combined Remodelin with the EGFR-specific monoclonal antibody cetuximab and tested its efficacy in CRC cells with wild-type KRAS/NRAS/BRAF. EGFR inhibition effectively augmented the therapeutic efficacy of Remodelin both in vitro and in vivo. These data support the potential value of dual NAT10/EGFR inhibition as a strategy to improve treatment response in patients with wild-type KRAS/NRAS/BRAF colorectal tumors.

Based on public datasets, we discovered that high NAT10 expression is significantly associated with a worse prognosis in KRAS-mutant CRC and is correlated with a better prognosis in KRAS wild-type CRC. Why is there a significant difference in the prognostic value of NAT10 in these two types of CRC patients? We speculate that it may be related to the activation of ERRFI1, an EGFR inhibitor mediated by NAT10. Due to the shortage of clinical specimens with KRAS mutation information, we were unable to verify these results. Further studies utilizing large-scale clinical datasets are required to explore the prognostic value of NAT10 in patients with different genetic backgrounds. Moreover, we postulate that the degree of the cancer-promoting impact of NAT10 in CRC might be related to the mutation status of KRAS. Specifically, NAT10 may exert a stronger cancer-promoting effect in KRAS mutant cells compared to KRAS wild-type cells. In KRAS mutant cells, the EGFR signaling pathway is constantly activated. Although ERRFI1 can be activated by NAT10 to inhibit EGFR, this inhibition has minimal impact on down-regulating the downstream signaling pathway of EGFR in KRAS mutant cells. However, in KRAS wild-type cells, although NAT10 has an activating effect on the PI3K-AKT pathway, NAT10 also inhibits the downstream EGFR signaling pathway by activating ERRFI1, which to a certain extent counteracts the activated PI3K-AKT pathway. Consequently, the cancer-promoting effect of NAT10 in KRAS wild-type cells is relatively weak. However, our study found that there is no significant positive correlation between NAT10 basal expression and the therapeutic efficacy of Remodelin. This may be related to the expression levels of ERRFI1 and EGFR in CRC cells and warrants further investigation in the future.

According to prior studies, the NAT10-mediated ac4C modification of RNA facilitates cancer cells’ adaptation to drugs or other extracellular stimuli [7, 22]. Consequently, we examined changes in NAT10 expression patterns under various stimuli, including heat shock, chemotherapy, and oxidative stress. Interestingly, 5-Fu treatment promoted the interaction between NAT10 and UBR5, enhancing the ubiquitin-mediated degradation of NAT10. In other words, 5-Fu treatment may induce EGFR reactivation in a NAT10-ERRFI1 axis-dependent manner. We propose that combining an EGFR inhibitor and 5-Fu may be beneficial for advanced CRC, in line with recent clinical trials demonstrating that adding panitumumab, another anti-EGFR antibody, to standard first-line chemotherapy (mFOLFOX6) significantly improved overall survival in patients with unresectable, RAS wild-type, left-sided, metastatic CRC, compared to adding an anti-VEGF antibody (bevacizumab) to mFOLFOX6 [23]. Moreover, we observed that the combination of 5-Fu with cetuximab and Remodelin increased tumor regression in our xenograft mouse model of wild-type KRAS/NRAS/BRAF CRC. Although it remains unclear whether such a triple combination therapy would benefit the broader population of patients with RAS wild-type metastatic CRC, this warrants investigation in future clinical trials.

Some limitations and unresolved questions should be addressed in future research. For example, in studying combination regimens to overcome Remodelin resistance, we only employed subcutaneous xenograft mouse models to evaluate therapeutic effects; additional models should be assessed to corroborate our conclusions. While EGFR inhibition effectively enhanced the efficacy of Remodelin, and triple combination therapy (5-FU, Remodelin, and cetuximab) promoted tumor regression in our xenograft mouse models with wild-type KRAS/NRAS/BRAF CRC, the potential side effects of this combined treatment strategy must be considered. Therefore, it is essential to identify a therapeutic window that optimizes CRC cell eradication while minimizing adverse effects on healthy cells for the clinical application of NAT10 inhibitors. In this study, we mainly explored the alterations in NAT10 expression patterns under diverse stimuli including heat shock, chemotherapy, and oxidative stress. Ultimately, we discovered that 5-Fu treatment enhanced the interaction between NAT10 and UBR5 and augmented the ubiquitin-mediated degradation of NAT10. Regarding the low expression of NAT10 in normal colon tissues as shown in our data, we fully acknowledge that whether the low levels of NAT10 in these cells are maintained by the ubiquitin-proteasome system remains uncertain. More investigations are required for further exploration to understand its overall role in colorectal biology and detemine the influence of KRAS mutation on NAT10 expression. Additionally, we will also consider exploring other possible regulatory mechanisms that may be involved in NAT10 expression regulation, such as transcriptional regulation and post-translational modifications other than ubiquitination.

## Conclusion

In conclusion, we demonstrated the crucial physiological functions of the NAT10-mediated ac4C modification of ERRFI1 in Remodelin resistance. The low response rate and resistance hinder the wide clinical application of Remodelin. Our study provides a theoretical basis for the development of novel therapeutic strategies to improve Remodelin sensitivity and efficacy in cancer therapy.

## Electronic supplementary material

Below is the link to the electronic supplementary material.

Supplementary Material 1: Additional file 1: Supplementary Table 1. Reagents used in the study.

Supplementary Material 2: Additional file 2: Supplementary Table 2. Antibodies used in the study.

Supplementary Material 3: Additional file 3: Supplementary Table 3. Primers used in this study.

Supplementary Material 4: Additional file 4: Supplementary Table 4. Mass spectrometry identification of NAT10 ubiquitination sites.

Supplementary Material 5: Additional file 5: Supplementary Figure 1. NAT10 promotes proliferation and inhibits apoptosis of CRC cells in vitro. (A) Knockdown transfection efficiency of NAT10 in SW620 and SW48 cells determined using WB. (B-C) Effects of NAT10 on proliferation were measured using the EdU assay (B) and colony formation assays (C). (D) Flow cytometry was used to detect rates of apoptosis (LR+UR) of indicated cells. (E) The cell cycle distribution was detected by flow cytometry in NAT10 knockdown cells. The data are representative of three independent experiments and presented as the mean ± SD. Comparisons were performed using two-tailed unpaired Student’s t-tests. *P <0.05, **P < 0.01, ***P < 0.001, ****P < 0.0001

Supplementary Material 6: Additional file 6: Supplementary Figure 2. ERRFI1 was regulated by NAT10 and participated in CRC malignant progression. (A) ERRFI1 were detected by WB in the indicated cells. (B) The effects of ERRFI1 on CRC cell proliferation were measured using an EdU assay. (C) Flow cytometry was performed to detect the apoptotic rate (LR + UR) of the indicated cells. (D) Cell cycle distribution was detected using flow cytometry in ERRFI1 knockdown cells. The data are representative of three independent experiments and presented as the mean ± SD. *P < 0.05, **P < 0.01, ***P < 0.001, as determined using two-tailed unpaired Student’s t-tests.

Supplementary Material 7: Additional file 7: Supplementary Figure 3. Schematic diagram of the ERRFI1 plasmids. (A) PACES tools (http://rnanut.net/paces/) were used to predict conserved acetylation sites in the *ERRFI1* CDS. (B) Schematic representation of one wild-type and five mutant HA-labeled *ERRFI1* plasmids. Cytosine (C) was replaced with thymine (T) in the potential ac4C peak of the mutant *ERRFI1* 5’-UTR or CDS region.

Supplementary Material 8: Additional file 8: Supplementary Figure 4. UBR5 mediates NAT10 ubiquitination upon 5-Fu treatment. (A-B) WB analysis of NAT10 protein expression in CRC cell lines. (C) WB analysis of NAT10 and ERRFI1 expression in CRC cell lines. (D) WB analysis of NAT10 and ERRFI1 expression in DLD1-LV-NAT10 cells treated with indicated drugs. (E) WB analysis of NAT10 and ERRFI1 expression in SW620 cells treated with indicated drugs. (F) GO enrichment analysis of NAT10-interacting proteins identified by mass spectrometry of NAT10-specific complexes from SW620 cells. Colors from red to blue indicates a decrease in the P-value. (G-H) Knockdown transfection efficiency of UBR5 in DLD1 (G) and SW620 (H) cells determined using WB analysis. (I-J) WB analysis of NAT10 in indicated cells treated or not treated with 5-Fu. (K) Mass spectrometry identification of K195, K262, K274, K426 and K520 ubiquitination of NAT10.

Supplementary Material 9: Additional file 9: Supplementary Figure 5. Conservative analysis of NAT10 among different species.

## Data Availability

RNA-seq and acRIP-seq, as well as RIP-seq data have been deposited in the GEO database (GSE265992 https://www.ncbi.nlm.nih.gov/geo/query/acc.cgi? acc=GSE265992; GSE265993. https://www.ncbi.nlm.nih.gov/geo/query/acc.cgi? acc=GSE265993; GSE264582 https://www.ncbi.nlm.nih.gov/geo/query/acc.cgi? acc=GSE264582). The other data used and/or analyzed during the current study are available from the corresponding author on reasonable request.

## Abbreviations

CRC: Colorectal cancer
NAT10: N-acetyltransferase 10
MIG6: Mitogen-inducible gene 6
ERRFI1: ERBB receptor feedback inhibitor 1
EGFR: Epidermal growth factor receptor
p-EGFR: Phospho-EGF receptor antibody
PI3K: Phosphatidylinositol-3-kinase
mTOR: Mammalian target of rapamycin
AKT: Serine/threonine kinase
p-AKT: Phospho-AKT (Ser473) antibody
ERK1/2: Extracellular-regulated kinase 1/2
p-ERK1/2: Phospho-extracellular-regulated kinase 1/2
STAT1: Signal transducer and activator of transcription 1
p-STAT1: Phospho-signal transducer and activator of transcription 1
UBR5: Ubiquitin protein ligase E3 component n-recognin 5
3-MA: 3-methyladenine
CQ: Chloroquine
CHX: Cycloheximide
ACTD: Actinomycin D
EdU: 5-ethynyl-2’-deoxyuridine
KRAS: Kirsten rats arcomaviral oncogene homology
