# Supplementary material for: EGFR inhibition augments the therapeutic efficacy of the NAT10 inhibitor Remodelin in Colorectal cancer
Source: J Exp Clin Cancer Res. 2025 Feb 4;44:37. doi: 10.1186/s13046-025-03277-y (PMC11792579; doi:10.1186/s13046-025-03277-y)
Supplement: Supplementary file 1 — Supplementary Material 1: Additional file 1: Supplementary Table 1. Reagents used in the study. [file 13046_2025_3277_MOESM1_ESM.docx]

**Supplementary Table 1**

| Reagent | Company |
| --- | --- |
| DMSO | China, Aladdin, CAS# 67-68-5 |
| 5-FU | USA, MedChem Express, HY-16706 |
| Remodelin | USA, MedChem Express, HY-90006 |
| Cetuximab | USA, MedChem Express, HY-P9905 |
| Puromycin | China, Biosharp, BL528A |
| Penicillin&Streptomycin Solution | China, Biosharp, BL505A |
| Leibovitz's L-15 | China, Biosharp, BL313A |
| RPMI 1640 | China, Biosharp, BL303A |
| Dulbecco's Modified Eagle Medium (DMEM) | China, Biosharp, BL301A |
| Minimum Essential Medium (MEM) | China, Pricella, PM150410C |
| Fetal Bovine Serum | USA, Thermo Fisher Scientific, CAS#10100147 |
| Trizol | USA, Thermo Fisher Scientific, CAS#A33250 |
| Actinomycin D (ACTD) | USA, MedChem Express, HY-17559 |
| 3-Methyladenine (3-MA) | USA, Selleck Chemicals, S2767 |
| Chloroquine (CQ) | USA, Selleck Chemicals, S6999 |
| Z-Leu-Leu-Leu-al (MG132) | USA, Selleck Chemicals, S2619 |
| Cycloheximide (CHX) | USA, Selleck Chemicals, S7418 |
| Polyethylene Glycol 300 (PG300) | USA, Selleck Chemicals, S6704 |
| Tween 20 | USA, Selleck Chemicals, S6702 |
| Transfer Buffer | USA, BIO-RAD, Cat#10026938 |
| Nitrocellulose membrane | USA, BIO-RAD, Cat#1620115 |
| TGX Fast Cast Acrylamide Kit | USA, BIO-RAD, Cat#1610183 |
| SDS-PAGE Running Buffer | China, Servicebio(Wuhan), G2081 |
| TBST | China, Servicebio(Wuhan), G0004 |
| PBS | China, Servicebio(Wuhan), G4202 |
| Paraformaldehyde | China, Servicebio(Wuhan), G1101 |
| Skimmed Milk Powder | China, Servicebio(Wuhan), G310001 |
| Primary Antibody Dilution Buffer | China, Servicebio(Wuhan), G2025 |
| Methylene Blue | China, Aladdin, CAS# 28983-56-4 |
| ECL Reagent | China, Epizyme Biomedical, SQ202L |
| Protein Marker | China, Epizyme Biomedical, WJ103 |
| Cell Counting Kit-8 | China, Biosharp, BL1055 |
| [5-ethynyl-2'-deoxyuridine](https://www.yeasen.com/products/detail/1556) Kit (EdU) | China, Beyotime, C0075S |
| AnnexinV-APC7-AAD Apoptosis Kit | China, Multi Sciences, AP-60-PCS |
| PrimeScript™ RT Reagent Kit | Japan, Takara, Cat# RR036A |
| Quantitative Real-time PCR Kit | Japan, Takara, Cat# RR820A |
| HRP-labeled Goat Anti-Rabbit IgG | China, ZhongShan Golden Bridge, ZB-2301 |
| Universal Two-Step Assay Kit | China, Zhongshan Golden Bridge(Beijing), SP-9000 |
| Diaminobenzidine Kit (DAB) | China, Zhongshan Golden Bridge(Beijing), ZLI-9019 |
| Protein Quantification Kit | USA, Thermo Fisher Scientific, BL1054A |
| Ac4C-RIP-seq Kit | China, Cloud-seq(Shanghai), GS-ET-005 |
| Immunoprecipitation Kit | USA, Abcam, Cat#ab206996 |
| Lipo8000 | China, Beyotime, C0533 |
| EndoFree Mini Plasmid Kit II | China,TIANGEN, DP118-02 |
| Ampicillin | China, Servicebio(Wuhan), GC301004 |
| SerRed | China, Servicebio(Wuhan), G3606 |
| LB | China, Servicebio(Wuhan), G3103 |
| 1×TAE | China, Servicebio(Wuhan), G3374 |
| 6×DNA Loading Buffer | China, Servicebio(Wuhan), G3011 |
| [Agarose](https://www.servicebio.cn/search-result?search=G5056) | China, Servicebio(Wuhan), GC205013 |
| Prestained Protein Marker | China, Servicebio(Wuhan), G2089 |
