# Supplementary material for: EGFR inhibition augments the therapeutic efficacy of the NAT10 inhibitor Remodelin in Colorectal cancer
Source: J Exp Clin Cancer Res. 2025 Feb 4;44:37. doi: 10.1186/s13046-025-03277-y (PMC11792579; doi:10.1186/s13046-025-03277-y)
Supplement: Supplementary file 2 — Supplementary Material 2: Additional file 2: Supplementary Table 2. Antibodies used in the study. [file 13046_2025_3277_MOESM2_ESM.docx]

Supplementary Table 2

| Antibody | Company | Dilution Information |
| --- | --- | --- |
| GAPDH | UK, Abcam, Cat#60004-1-Ig | (1:10000; Mouse; 36kD) |
| ACTIN | China, Proteintech, Cat#23660-1-AP | (1:2000; Mouse; 42kD) |
| TUBULIN | China, Proteintech, Cat#11224-1-AP | (1:5000; Rabbit; 55kD) |
| NAT10 | UK, Abcam, Cat#ab194297 | (1:2000; Rabbit; 116kD) |
| PI3K | China, Abmart, Cat#T40115 | (1:2000; Rabbit; 85kD) |
| p-PI3K | China, Abmart, Cat#[T40116](http://www.ab-mart.com.cn/page.aspx?node=%2077%20&id=%2049664) | (1:2000; Rabbit; 85kD) |
| mTOR | China, Abmart, Cat#[T55306](http://www.ab-mart.com.cn/page.aspx?node=%2077%20&id=%201583) | (1:1000; Rabbit; 289kD) |
| p-mTOR | China, Abmart, Cat#[T56571](http://www.ab-mart.com.cn/page.aspx?node=%2077%20&id=%202240) | (1:1000; Rabbit; 289kD) |
| ERRFI1 | USA, Cell Signaling Technology, Cat#2440 | (1: 500; Rabbit; 51kD) |
| EGFR | USA, Cell Signaling Technology, Cat#4267 | (1:4000; Rabbit; 145kD) |
| p-EGFR | USA, Cell Signaling Technology, Cat#4407 | (1:1000; Rabbit; 175kD) |
| AKT | USA, Cell Signaling Technology, Cat#4685 | (1:2000; Rabbit; 56kD) |
| p-AKT | UK, Abcam, Cat#ab205718 | (1:2000; Rabbit; 56kD) |
| ERK1/2 | USA, Cell Signaling Technology, Cat#4695 | (1:1000; Rabbit; 44&42kD) |
| p-ERK1/2 | USA, Cell Signaling Technology, Cat#4370 | (1:2000; Rabbit; 44&42kD) |
| STAT1 | China, Proteintech, Cat#10144-2-AP | (1:2000; Rabbit; 84kD) |
| P-STAT1 | China, Proteintech, Cat#28977-1-AP | (1:1000; Rabbit; 84kD) |
| UBR5 | USA, Cell Signaling Technology, Cat#65344 | (1:1000; Rabbit; 300kD) |
| Ub | China, Proteintech, Cat#10201-2 | (1: 600; Rabbit; 26kD) |
| Ki67 | China, Proteintech, Cat#28074-1-AP | (1: 250; Rabbit; 351kD) |
| FLAG | China, Servicebio, Cat#20543-1-AP | (1: 5000; Rabbit) |
| HA | China, Proteintech, Cat#51064-2-AP | (1: 5000; Rabbit) |
| MYC | China, Servicebio, Cat#GB12076-100 | (1: 5000; Mouse) |
