# Supplementary material for: EGFR inhibition augments the therapeutic efficacy of the NAT10 inhibitor Remodelin in Colorectal cancer
Source: J Exp Clin Cancer Res. 2025 Feb 4;44:37. doi: 10.1186/s13046-025-03277-y (PMC11792579; doi:10.1186/s13046-025-03277-y)
Supplement: Supplementary file 3 — Supplementary Material 3: Additional file 3: Supplementary Table 3. Primers used in this study. [file 13046_2025_3277_MOESM3_ESM.docx]

**Supplementary Table 3**

| Target | Sequence |
| --- | --- |
| ACTIN-F | CGTGACATTAAGGAGAAGCTG |
| ACTIN-R | CTAGAAGCATTTGCGGTGGAC |
| NAT10-F | CTGCTGAGAATAAGACCACGACGA |
| NAT10-R | TTGAGGCAATCCAGGCACAG |
| ERRFI1-F | TGAGGAAGACCTACTGGAGCAG |
| ERRFI1-R | GTATTAGGCGCTCCTGAGCAGA |
| UBR5-F | CCAGACAGATTGGAATTGGGTAA |
| UBR5-R | CATGGAGAGTCGCTTGTCCT |
| HA-F | CTGCGGTATATCTTCAGCCACA |
| HA-R | CATAAGGGTATGATCCTCCACCT |
