# Supplementary figures and images for: EGFR inhibition augments the therapeutic efficacy of the NAT10 inhibitor Remodelin in Colorectal cancer
Source: J Exp Clin Cancer Res. 2025 Feb 4;44:37. doi: 10.1186/s13046-025-03277-y (PMC11792579; doi:10.1186/s13046-025-03277-y)

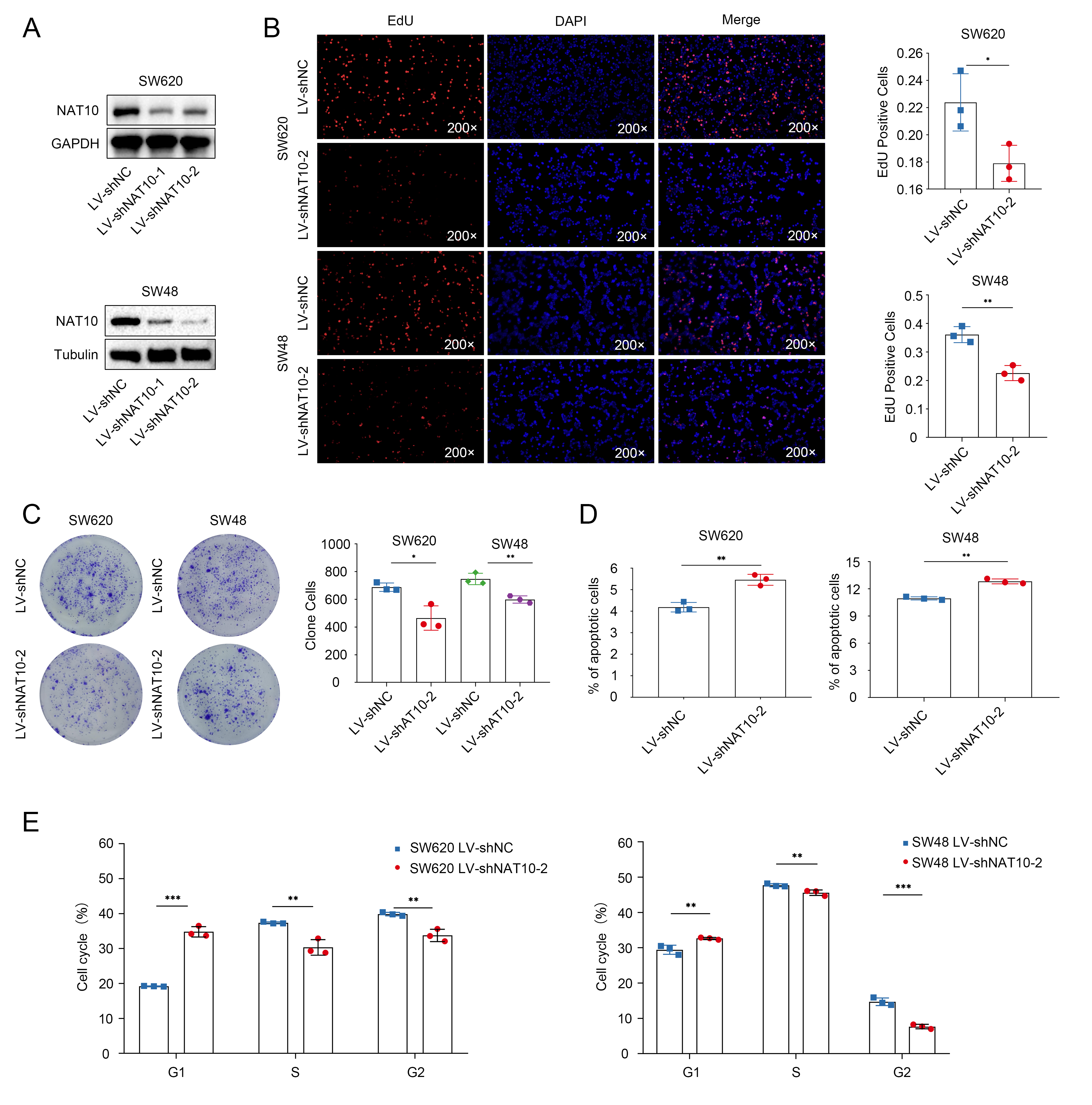

Supplement: Supplementary file 5 — Supplementary Material 5: Additional file 5: Supplementary Figure 1. NAT10 promotes proliferation and inhibits apoptosis of CRC cells in vitro. (A) Knockdown transfection efficiency of NAT10 in SW620 and SW48 cells determined using WB. (B-C) Effects of NAT10 on proliferation were measured using the EdU assay (B) and colony formation assays (C). (D) Flow cytometry was used to detect rates of apoptosis (LR+UR) of indicated cells. (E) The cell cycle distribution was detected by flow cytometry in NAT10 knockdown cells. The data are representative of three independent experiments and presented as the mean ± SD. Comparisons were performed using two-tailed unpaired Student’s t-tests. *P <0.05, **P < 0.01, ***P < 0.001, ****P < 0.0001 [file 13046_2025_3277_MOESM5_ESM.tif]

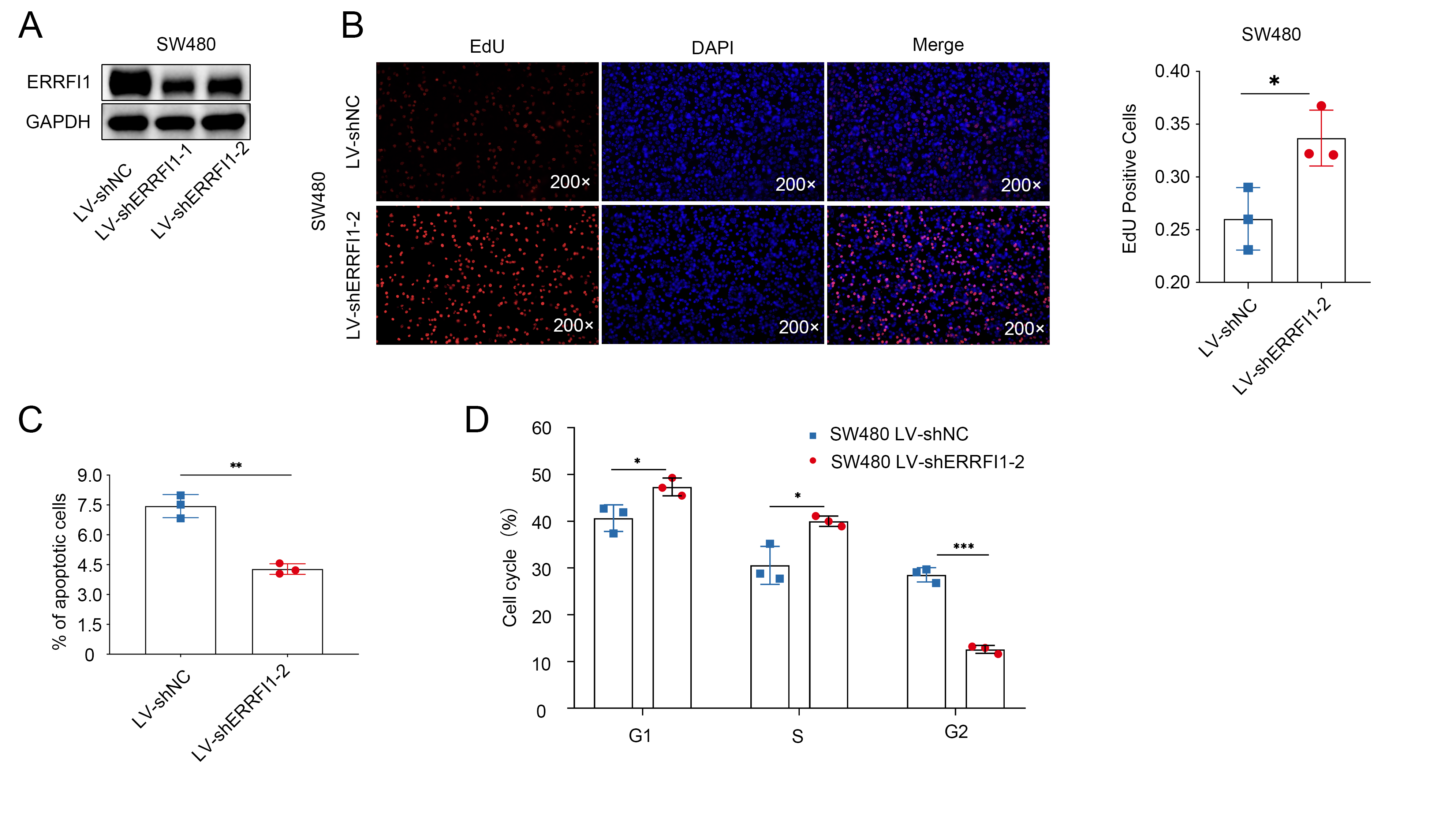

Supplement: Supplementary file 6 — Supplementary Material 6: Additional file 6: Supplementary Figure 2. ERRFI1 was regulated by NAT10 and participated in CRC malignant progression. (A) ERRFI1 were detected by WB in the indicated cells. (B) The effects of ERRFI1 on CRC cell proliferation were measured using an EdU assay. (C) Flow cytometry was performed to detect the apoptotic rate (LR + UR) of the indicated cells. (D) Cell cycle distribution was detected using flow cytometry in ERRFI1 knockdown cells. The data are representative of three independent experiments and presented as the mean ± SD. *P < 0.05, **P < 0.01, ***P < 0.001, as determined using two-tailed unpaired Student’s t-tests. [file 13046_2025_3277_MOESM6_ESM.tif]

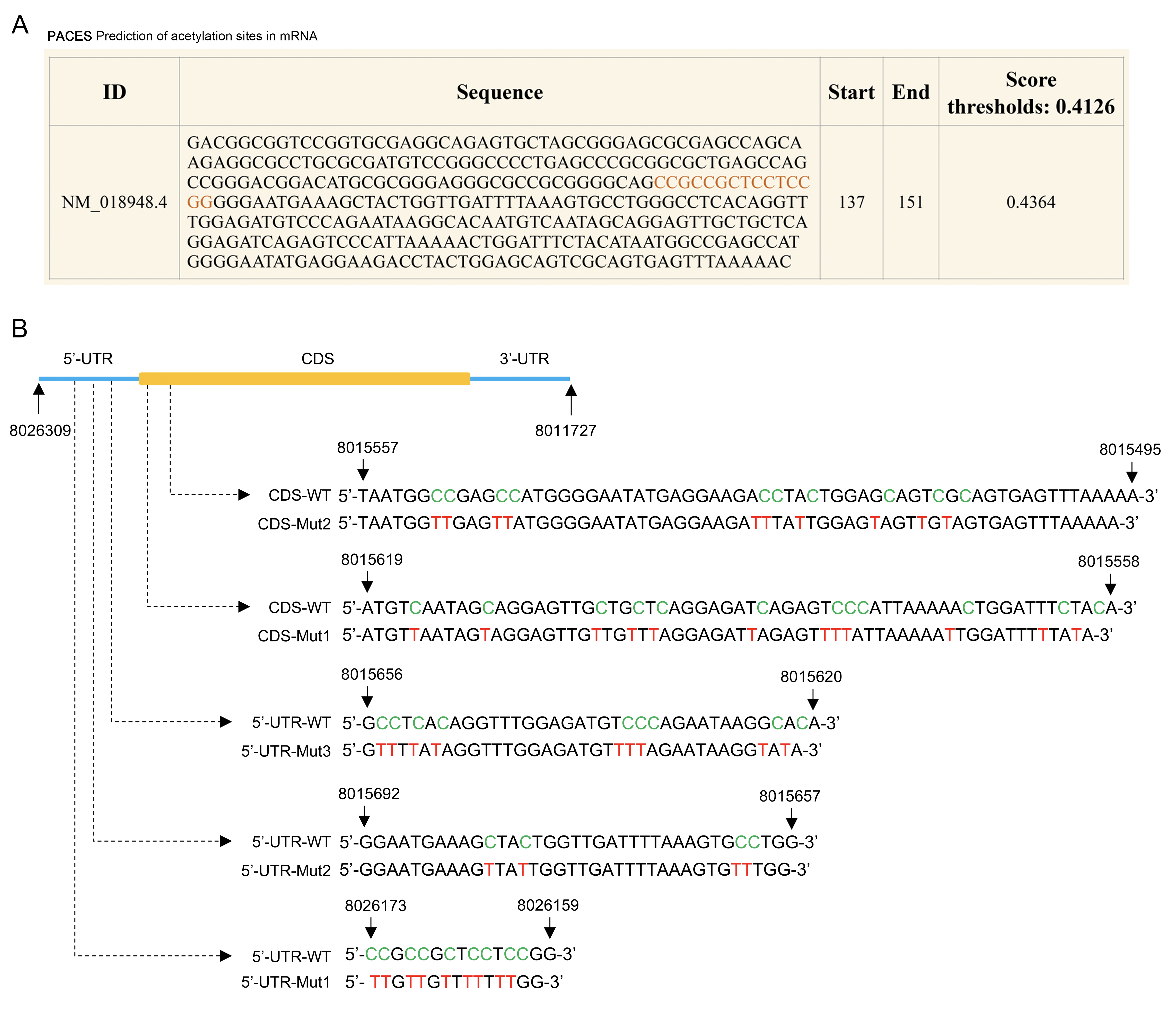

Supplement: Supplementary file 7 — Supplementary Material 7: Additional file 7: Supplementary Figure 3. Schematic diagram of the ERRFI1 plasmids. (A) PACES tools (http://rnanut.net/paces/) were used to predict conserved acetylation sites in the ERRFI1 CDS. (B) Schematic representation of one wild-type and five mutant HA-labeled ERRFI1 plasmids. Cytosine (C) was replaced with thymine (T) in the potential ac4C peak of the mutant ERRFI1 5’-UTR or CDS region. [file 13046_2025_3277_MOESM7_ESM.tif]

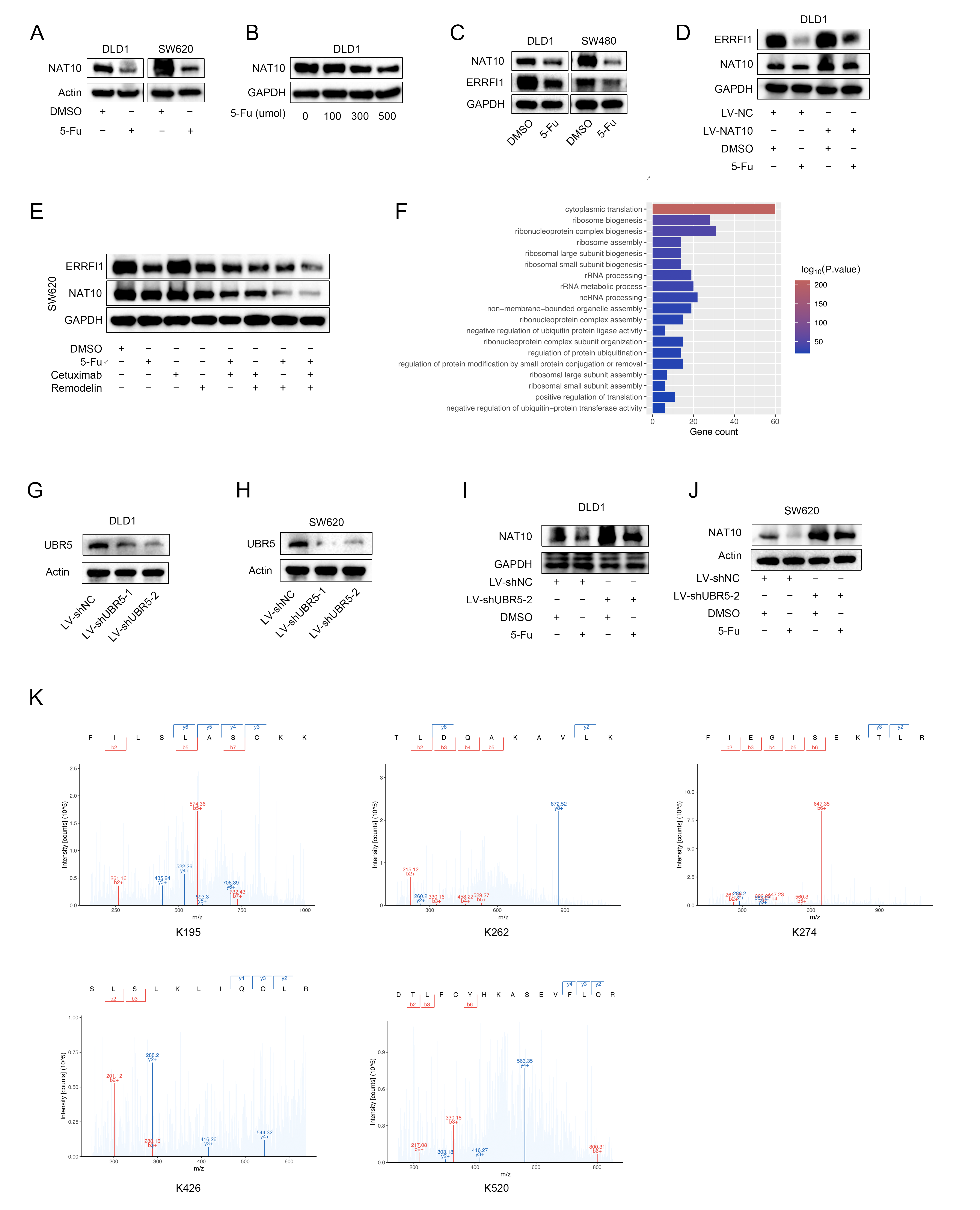

Supplement: Supplementary file 8 — Supplementary Material 8: Additional file 8: Supplementary Figure 4. UBR5 mediates NAT10 ubiquitination upon 5-Fu treatment. (A-B) WB analysis of NAT10 protein expression in CRC cell lines. (C) WB analysis of NAT10 and ERRFI1 expression in CRC cell lines. (D) WB analysis of NAT10 and ERRFI1 expression in DLD1-LV-NAT10 cells treated with indicated drugs. (E) WB analysis of NAT10 and ERRFI1 expression in SW620 cells treated with indicated drugs. (F) GO enrichment analysis of NAT10-interacting proteins identified by mass spectrometry of NAT10-specific complexes from SW620 cells. Colors from red to blue indicates a decrease in the P-value. (G-H) Knockdown transfection efficiency of UBR5 in DLD1 (G) and SW620 (H) cells determined using WB analysis. (I-J) WB analysis of NAT10 in indicated cells treated or not treated with 5-Fu. (K) Mass spectrometry identification of K195, K262, K274, K426 and K520 ubiquitination of NAT10. [file 13046_2025_3277_MOESM8_ESM.tif]
