# Supplementary material for: EGFR inhibition augments the therapeutic efficacy of the NAT10 inhibitor Remodelin in Colorectal cancer
Source: J Exp Clin Cancer Res. 2025 Feb 4;44:37. doi: 10.1186/s13046-025-03277-y (PMC11792579; doi:10.1186/s13046-025-03277-y)
Supplement: Supplementary file 9 — Supplementary Material 9: Additional file 9: Supplementary Figure 5. Conservative analysis of NAT10 among different species. [file 13046_2025_3277_MOESM9_ESM.docx]

Supplementary Figure 5. Conservative analysis of NAT10 among different species
